# Twisted-layer boron nitride ceramic with high deformability and strength

**DOI:** 10.1038/s41586-024-07036-5

**Published:** 2024-02-21

**Authors:** Yingju Wu, Yang Zhang, Xiaoyu Wang, Wentao Hu, Song Zhao, Timothy Officer, Kun Luo, Ke Tong, Congcong Du, Liqiang Zhang, Baozhong Li, Zewen Zhuge, Zitai Liang, Mengdong Ma, Anmin Nie, Dongli Yu, Julong He, Zhongyuan Liu, Bo Xu, Yanbin Wang, Zhisheng Zhao, Yongjun Tian

**Affiliations:** 1 Center for High Pressure Science (CHiPS), State Key Laboratory of Metastable Materials Science and Technology, Yanshan University, Qinhuangdao, China; 2 Center for Advanced Mechanics and Materials, Applied Mechanics Laboratory, Department of Engineering Mechanics, Tsinghua University, Beijing, China; 3 School of Materials Science and Engineering, Shaanxi University of Technology, Hanzhong, China; 4 Center for Advanced Radiation Sources, The University of Chicago, Chicago, IL USA; 5 Clean Nano Energy Center, State Key Laboratory of Metastable Materials Science and Technology, Yanshan University, Qinhuangdao, China

**Keywords:** Ceramics, Mechanical properties

## Abstract

Moiré superlattices formed by twisted stacking in van der Waals materials have emerged as a new platform for exploring the physics of strongly correlated materials and other emergent phenomena^1–5^. However, there remains a lack of research on the mechanical properties of twisted-layer van der Waals materials, owing to a lack of suitable strategies for making three-dimensional bulk materials. Here we report the successful synthesis of a polycrystalline boron nitride bulk ceramic with high room-temperature deformability and strength. This ceramic, synthesized from an onion-like boron nitride nanoprecursor with conventional spark plasma sintering and hot-pressing sintering, consists of interlocked laminated nanoplates in which parallel laminae are stacked with varying twist angles. The compressive strain of this bulk ceramic can reach 14% before fracture, about one order of magnitude higher compared with traditional ceramics (less than 1% in general), whereas the compressive strength is about six times that of ordinary hexagonal boron nitride layered ceramics. The exceptional mechanical properties are due to a combination of the elevated intrinsic deformability of the twisted layering in the nanoplates and the three-dimensional interlocked architecture that restricts deformation from propagating across individual nanoplates. The advent of this twisted-layer boron nitride bulk ceramic opens a gate to the fabrication of highly deformable bulk ceramics.

## Abstract (web summary)

A bulk ceramic composed of interlocked boron nitride nanoplates with a laminated structure of twist-stacked nanoslices is created using hot-pressing and spark plasma sintering, which exhibits large elastic and plastic deformability and high strength at room temperature.

## Main

Moiré superlattices formed by stacking layered van der Waals (vdW) crystalline sheets with slight relative rotations (twist angles) about the stacking direction have prompted intensive research efforts to explore strongly correlated physics. The introduction of twist angles breaks the inherent symmetry of the crystal structure and often causes unique changes in physical properties. Examples include superconductivity in magic-angle bilayer and multilayer graphene^1–3^ and ferroelectric-like domains originating at the interface between two marginally twisted hexagonal boron nitride (hBN) thin crystals^4^. Theoretical simulations predict that adjusting the twist angle in two-dimensional transition metal dichalcogenide may result in new physical phenomena such as spin-liquid states, the quantum anomalous Hall effect and chiral *d*-wave superconductivity^5^. In addition to these new physical phenomena, experimental observations have shown that mechanical properties can be modified by changing the twist angle of vdW layered materials. For example, twisted-bilayer MoS_2_ shows significantly reduced friction^6^, and microscale graphite can maintain superlubricity with a wide range of bicrystal twist angles^7^. These findings hint that in vdW ceramic materials such as BN, the introduction of twisting in layered structures may produce notable effects on the deformability and strength of the bulk ceramic.

hBN is a typical vdW material with a layered crystal structure^8^. At room temperature, bulk hBN ceramics, which are widely used in industry, are brittle with a low compressive strength of about 100 MPa and can sustain a very limited amount of elastic deformation (usually at the level of 1%) before catastrophic failure^9,10^. By adding various sintering aids or reinforcing phases, the strength of hBN ceramics can be improved to some extent, but with little improvement in deformability^9,10^. Experimentally, large room-temperature elasticity and plasticity have been observed in micro/nanoscale ceramic monocrystals and oligocrystals, such as diamond^11^, AlN^12^, TiO_2_ (ref. ^13^), Si_3_N_4_ (ref. ^14^) and ZrO_2_ (ref. ^15^). However, achieving similar room-temperature deformability in bulk dense ceramics remains a formidable challenge^16^. It was recently proposed that the deformability of an inorganic vdW material could be assessed on the basis of three key factors: a low slipping energy (*E*_s_) that allows interlayer gliding, a high cleavage energy (*E*_c_) that maintains interlayer integrity during gliding, and a suitable in-plane Young’s modulus (*Y*) that ensures intralayer flexibility^17,18^. These three parameters can be combined to define a deformability factor, *Ξ* = (*E*_c_/*E*_s_)(1/*Y*)^18^. In vdW materials, introducing twisted stacking may increase interlayer spacing^19^, which would reduce *E*_s_, thereby increasing the deformability factor^18^.

Here we report the synthesis of a bulk BN ceramic composed of three-dimensional interlocked BN nanoplates whose vdW layers form a laminated structure with various twisting angles. The twisted-layer bulk ceramic was synthesized from onion-like BN (oBN) nanoparticles using conventional spark plasma sintering (SPS) and hot-pressing sintering. This twisted-layer bulk ceramic shows exceptional deformability (up to 14% compressive strain) and plasticity (up to 8% permanent deformation) and high strength at room temperature. These remarkable properties have long been sought in engineering ceramics, which usually have very poor deformability with essentially no plasticity.

## Synthesis and microstructure

**Fig. 1:**
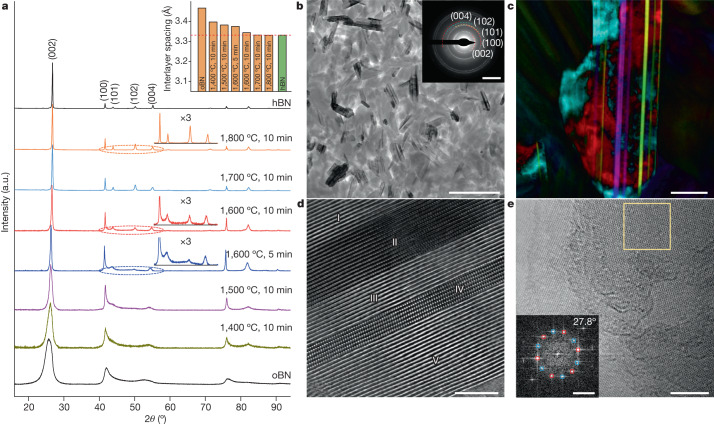
XRD patterns and microstructure of the bulk ceramics prepared through SPS. **a**, XRD patterns of bulk ceramics prepared under different SPS conditions. In some patterns, details of the circled regions are shown with intensity magnified by a factor of 3. Inset shows the interlayer spacing of prepared ceramics as a function of synthesis condition. **b**, Microstructure of the ceramic sintered at 1,600 °C for 5 min, showing randomly oriented nanoplates. Inset shows the corresponding SAED pattern, with hBN diffraction signals labelled. Extra diffraction halos and spots are present that do not belong to hBN. **c**, Differential phase contrast image of an edge-on nanoplate showing parallel nanoslices with different colours, indicating a laminated structure of BN nanoplates with parallel-stacked multiple BN nanoslices. **d**, HAADF-STEM image showing alternating regions of striped (I, III and V) and atomic (II and IV) resolution, evidencing differently twisted BN nanoslices in a laminated nanoplate. **e**, TEM image showing a moiré superlattice. The inset shows a fast Fourier transform pattern from the box region, where the rotational angle between two sets of diffraction spots (marked in red and blue, respectively) is 27.8°. Scale bars, 400 nm (**b**), 50 nm (**c**), 4 nm (**d**,**e**), 5 nm^−^^1^ (**b**,**e**, inset).

**Extended Data Fig. 1:**
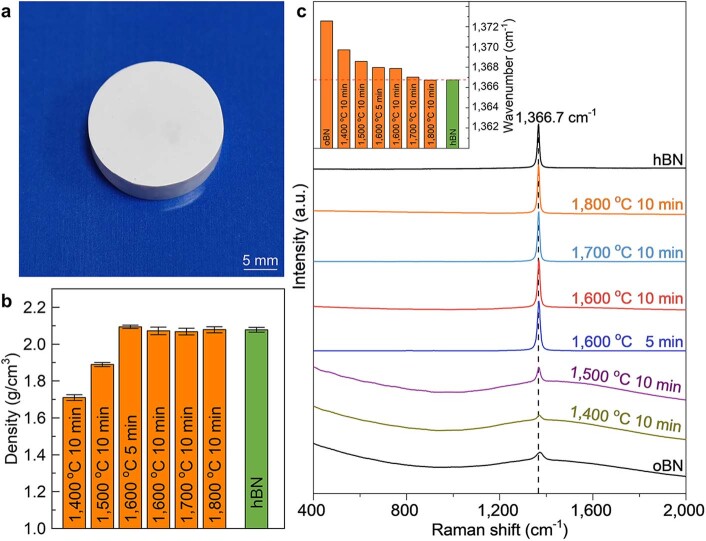
Density and Raman spectra of ceramics prepared by SPS sintering oBN under different conditions. **a**, Optical photo of the as-prepared ceramic block. **b**, Densities of sintered ceramics. Orange and green columns represent the ceramics prepared from oBN and hBN nanoprecursors, respectively. The sintering pressure was fixed at 50 MPa, and the sintering temperature and holding time were different, as shown in the figure. Error bars represent 1 s.d. (n  =  5). **c**, Raman spectra obtained from various samples. With increasing sintering temperature, the *E*_2g_ band of the as-prepared ceramics shifts toward low wavenumbers, approaching that of hBN. Inset compares the shift magnitude of this Raman band in wavenumber.

**Extended Data Fig. 2:**
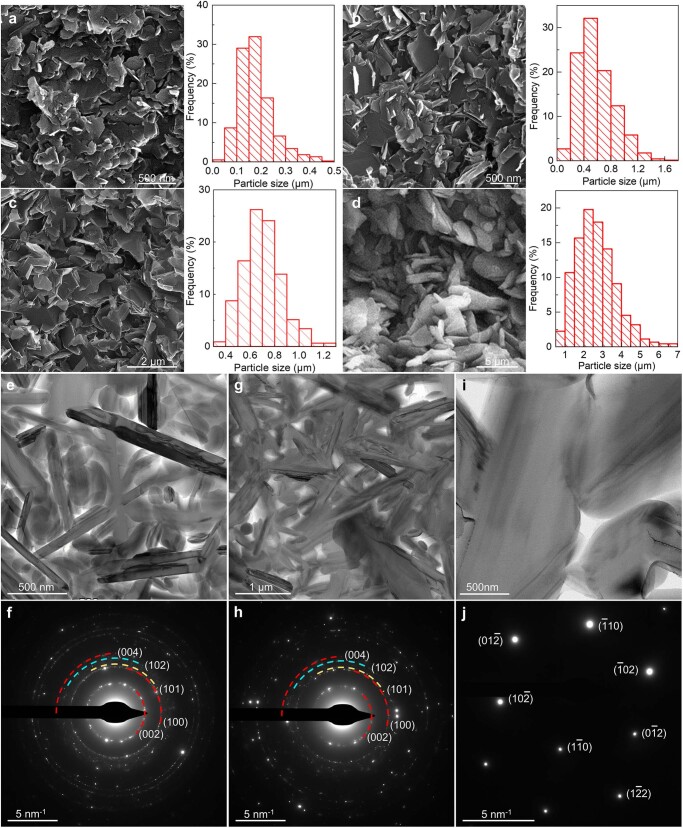
Morphology of fracture surface and microstructure of BN ceramics prepared by SPS sintering oBN under different conditions. **a**, 1,600 °C for 5 min. **b**, 1,600 °C for 10 min. **c**, 1,700 °C for 10 min. **d**, 1,800 °C for 10 min. The fracture surfaces were formed by directly breaking bulk ceramics with mechanical pliers. At 1,600 °C, the size of the nanoplates increases with dwelling time, from 5 min to 10 min. For a constant dwelling time of 10 min, the size of the nanoplates increases with temperature from 1,600 °C to 1,700 °C and 1,800 °C. The average lateral sizes of the nanoplates calculated from the corroded surface of the samples are *ca*. 173 nm, 583 nm, 704 nm, and 2.65 μm, respectively, in **a** through **d**. The STEM images and SAED patterns of the ceramics sintered at **e**, **f** 1,600 °C for 10 min, **g**, **h** 1,700 °C for 10 min, and **i**, **j** 1,800 °C for 10 min. With the increase of sintering temperature or time, the size of plates in ceramics increased from nanometer to micrometer. In **f** and **h**, the diffraction rings corresponding to different crystal planes of hBN were marked. There were additional new diffraction halos and dispersive diffraction spots between the (100) and (004) diffraction rings. In **j**, an ordinary hBN single crystal diffraction pattern taken from [221] zone axis was identified in the ceramic from a high sintering temperature of 1,800 °C.

oBN precursors are composed of turbostratic-nested BN spherical shells with abundant puckering and stacking faults^20,21^ (Supplementary Fig. 1). A series of bulk ceramics were sintered from oBN precursors by SPS. With increasing sintering temperature, the density of sintered ceramics increased until saturation at around 2.08 g cm^−^^3^ (Extended Data Fig. 1b). The original broad X-ray diffraction (XRD) peaks of oBN precursors gradually narrowed, and further peaks corresponding to hBN-like diffraction lines appeared, indicating a phase transition from oBN to an hBN-like layered structure (Fig. 1a). Under Raman spectroscopy, hBN-like characteristic Raman peaks developed with increasing temperature (Extended Data Fig. 1c). The ceramic sintered at 1,800 °C exhibited XRD patterns and Raman spectra identical to those of hBN ceramics, indicating a complete transition from oBN to hBN. However, XRD patterns of the ceramics sintered at 1,600 °C showed features different from those of hBN (Fig. 1a), for instance, larger interlayer spacing and higher background intensity between hBN (100) and (004) peaks. These differences were further confirmed by selected area electron diffraction (SAED) measurements (Fig. 1b, inset, and Extended Data Fig. 2f). Whereas the SAED patterns from the 1,800 °C sample were consistent with the standard crystallographic diffraction pattern of hBN (Extended Data Fig. 2j), the 1,600 °C sample exhibited pronounced halos and weak diffraction spots between hBN (100) and (004) diffraction rings. The weak spots did not belong to hBN, and hBN (101) and (102) rings were barely observed (Fig. 1b, inset, and Extended Data Fig. 2f,h). These observations imply that some metastable structures other than hBN were present in the ceramics sintered at 1,600 °C.

**Extended Data Fig. 3:**
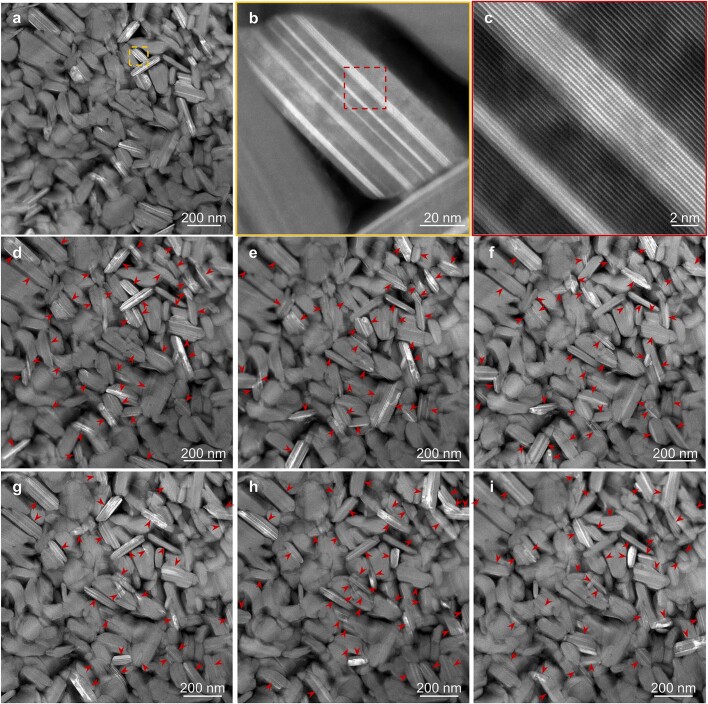
Ubiquitous presence of nanoplates with twisted-stacking structure in TS-BN-I ceramic. **a**-**c**, Gradually enlarged HAADF-STEM images. The bands with different contrasts in the nanoplate correspond to the laminated nanoslices with different in-plane orientations. **d**-**i**, HAADF-STEM images collected from the same region at different α-, β-tilt angles: **d** (2.5°, 15.2°), **e** (16.1°, 7.5°), **f** (9°, 3.1°), **g** (0°, 0°), **h** (−10°, −5°), and **i** (−15.5°, 10.2°). Red arrows indicate the appearance of nanoplates with bright and dark bands at different tilt angles, showing that nanoplates with twistedly stacking structure are ubiquitously present.

We investigated the microstructures of the SPS-sintered samples with scanning electron microscopy and scanning transmission electron microscopy (STEM). Ceramics sintered at temperatures between 1,600 and 1,800 °C were composed of plate-shaped grains (Fig. 1b and Extended Data Fig. 2). The average grain size increased with increasing sintering temperature. For the ceramic sintered at 1,600 °C for 5 min, the average thickness of the plates was approximately 40 nm and the lateral width was approximately 173 nm, close to the diameter of the oBN nanoprecursor. These nanoplates were randomly oriented, forming a three-dimensional nanoarchitecture (Fig. 1b), in striking contrast to the preferential oriented nanoplates routinely observed in polycrystalline ceramics sintered from hBN nanosheets (Supplementary Fig. 2). We characterized the detailed structure of the nanoplates in the sample sintered at 1,600 °C for 5 min. A differential phase contrast image of an edge-on nanoplate showed parallel straight bands in distinct colours (Fig. 1c), indicating a laminated structure with different laminae relatively rotated about the normal of the basal plane (that is, the lamination plane). High-angle annular dark-field (HAADF)-STEM observations along the zone axis of the nanoplate basal plane also showed narrow parallel regions in different (atomic or striped) resolutions, with thicknesses ranging from several to tens of atomic layers (I to V, Fig. 1d). These narrow parallel regions will be referred to as nanoslices hereafter. When the nanoplate was rotated around the normal of its basal plane (with the electron beam perpendicular to the rotation axis), transmission electron microscopy (TEM) images of different nanoslices moved in and out of atomic resolution (Supplementary Fig. 3). These observations indicate that the nanoplate consists of multiple nanoslices, sharing the same basal plane but twisted relative to one another by various angles around the normal of the basal plane. HAADF-STEM images collected from the same region at different tilt angles demonstrate that BN nanoplates composed of twist-stacked nanoslices are ubiquitously present in the bulk ceramic (Extended Data Fig. 3).

**Extended Data Fig. 4:**
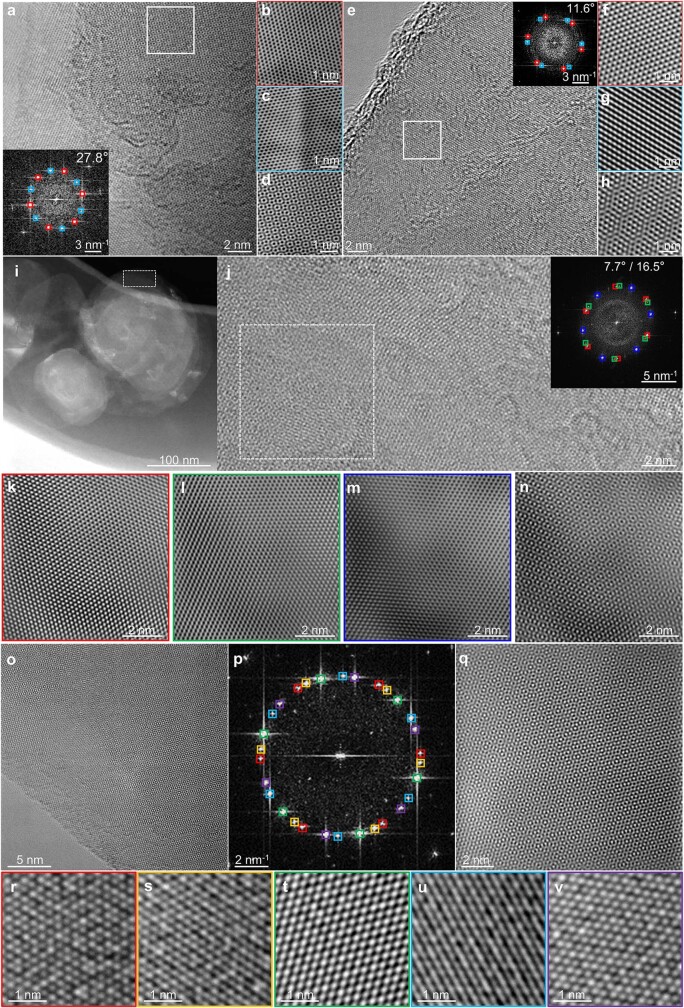
Moiré patterns formed by superimposing of two (a-h), three (i-n) and five (o-v) twisted nanoslices, respectively. **a** and **e**, HRTEM images taken at the edge of the nanoplate. The insets are the FFT patterns from white box areas in TEM images, showing two sets of superimposed diffraction patterns (marked with red and blue) with relative rotation angles of 27.8° and 11.6°, respectively. Note that **a** is the same as Fig. 1e. **b**, **c** and **f**, **g** Inverse FFT lattice images derived from FFT patterns. **d**, Moiré pattern formed by superimposing two sets of lattice images in **b** and **c**. **h**, Moiré pattern formed by superimposing two sets of lattice images in **f** and **g**. **i**, STEM dark field image of the nanoplate in ceramic. **j**, HRTEM image from the edge area (white dotted box) in **i**, showing the moiré pattern. Inset is the FFT pattern of white dotted box area in **j**, which is composed of three sets of diffraction spots. The adjacent twisted nanoslices are rotated by 7.7° and 16.5°, respectively. **k**, **l**, and **m** Inverse FFT lattice images derived from three sets of diffraction spots in FFT patterns marked with various colors. **n**, Moiré pattern formed by superimposing of three sets of lattice images in **k**, **l**, and **m**. **o**, HRTEM image taken from the edge of the nanoplate, showing the moiré pattern. **p**, FFT pattern superimposed by five sets of diffraction spots. **q**, Moiré pattern superimposed by five sets of lattice images in **r**-**v**. **r**-**v**, Inverse FFT lattice images derived from the five sets of diffraction spots in FFT pattern marked with various colors.

**Extended Data Fig. 5:**
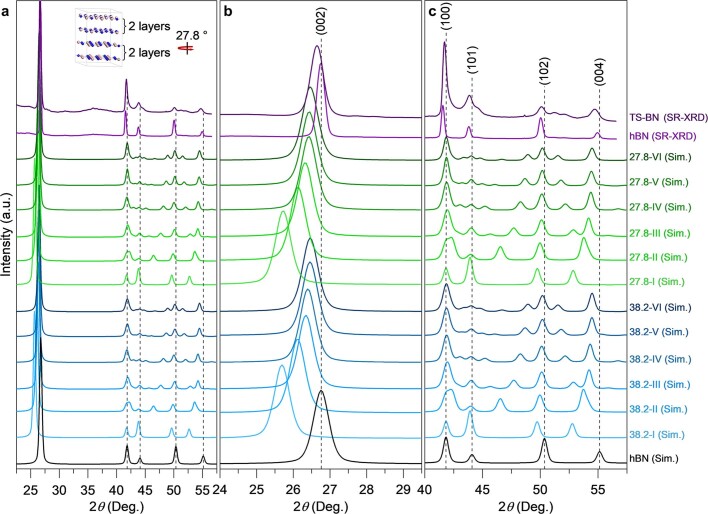
Simulated XRD patterns of hypothetical twist-stacked BN structures. **a**, Full patterns. Inset shows one twist-stacked structure, constructed by two-layer-thickness BN nanoslices stacked at a twist angle of 27.8°. **b** and **c**, Expanded patterns for details. Blue and green curves: crystal structures constructed by stacking nanoslices at a fixed twist angle (38.2°or 27.8°), where each nanoslice consists of 1 to 6 atomic layers (labeled as I through VI on the right-hand side, with twisting angle as prefix). Black curve: simulated XRD pattern of hBN structure. Purple curves: synchrotron radiation XRD (SR-XRD) patterns of as-prepared TS-BN and hBN ceramics measured at room temperature. The selected wavelength of X-ray for simulation was 1.54 Å. The van der Waals interaction between layers as well as interlayer spacings of the structures are corrected according to the difference between the simulated and measured interlayer spacings of hBN. Compared with hBN, the (002) diffraction peaks of twist-stacked structures shift to the left, indicating expanded interlayer spacings. Moreover, the twist-stacked structures possess additional diffraction peaks located between (100) and (004) diffraction lines of hBN. With increasing nanoslice thickness, these new diffraction peaks gradually approach to the (101) and (102) diffraction peaks of hBN. The result shows that the introduction of twist-stacked structures can account for the extra features in XRD patterns observed experimentally. The peak positions, Miller indices, and relative intensities of simulated diffraction peaks of hypothetical crystal structures are listed in Supplementary Table 1.

TEM observations along the normal of the nanoplate basal plane revealed a variety of moiré superlattices (Fig. 1e and Extended Data Fig. 4). The simulated moiré patterns fitted the experimental patterns well (Supplementary Fig. 4); however, the stacking of atomic layers in each nanoslice usually deviated from the AA′ stacking of ideal hBN, with frequent relative translation between neighbouring atomic layers (Fig. 1d). Furthermore, simulated results indicated that hypothetical structures formed by stacking twisted atomic layers had larger interlayer spacing than that of hBN and further diffraction peaks on both sides of hBN (101) and (102) peaks (Extended Data Fig. 5 and Supplementary Table 1). Thus, we interpret the extra features in our XRD and SAED observations (Fig. 1a,b and Extended Data Fig. 2) as due to in-plane twisting between adjacent nanoslices in the laminated structure.

The above observations show that the BN ceramic synthesized by SPS at 1,600 °C possesses a hierarchical microstructure. The honeycomb-structured BN atomic layers with identical orientation form individual nanoslices, which are then stacked with various in-plane twist angles to form laminated nanoplates. Finally, randomly oriented nanoplates form a three-dimensional nanoarchitecture of the bulk ceramic. Hereafter, we term this BN ceramic a TS-BN ceramic to emphasize the fact that laminated nanoplates are formed by twist-stacked nanoslices.

## Exceptional mechanical properties

**Fig. 2:**
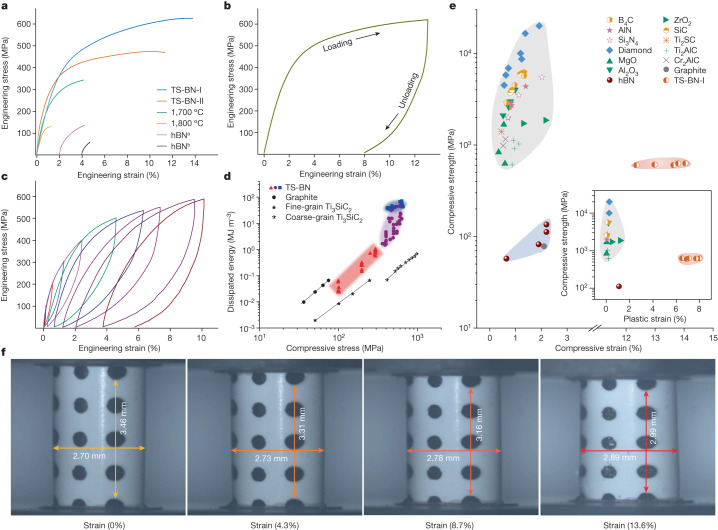
Superhigh room-temperature deformability and strength of TS-BN ceramic prepared through SPS. **a**, Engineering stress–strain curves. TS-BN-I sintered at 1,600 °C for 5 min exhibited an extraordinary high engineering strain of 14% and strength of 626 MPa, far exceeding those of ordinary hBN ceramics. TS-BN-II was prepared through SPS at 1,600 °C for 10 min. hBN^a^ and hBN^b^ are an SPS-sintered ceramic from hBN nanosheets and a commercial hBN ceramic, respectively. **b**, A single cyclic compression test on TS-BN-I, showing a permanent plastic deformation of 8% after unloading. **c**, Multiple cyclic test showing that TS-BN-I ceramic can sustain multiple loading–unloading cycles without fracture. **d**, log–log plot of dissipated energy versus uniaxial compressive stress. The red, purple and blue regions represent anelastic, plastic and load-to-failure stages, respectively. Results from polycrystalline graphite and Ti_3_SiC_2_ ceramics are included for comparison^22,23^. **e**, Comparison of uniaxial deformability and strength of TS-BN-I ceramic with those of traditional ceramics. Inset compares plastic deformability and strength of various ceramics. Detailed data are shown in Supplementary Table 2. **f**, Snapshots from a uniaxial compression test. Thin nickel marks are evaporated onto the specimen surface, serving as strain markers. See Supplementary Video 1 for the entire process.

Room-temperature uniaxial compression tests demonstrated exceptional mechanical properties of TS-BN bulk ceramics. Figure 2a summarizes the engineering stress–strain relationship until final fracture for different SPS-sintered BN samples. TS-BN sintered at 1,600 °C for 5 min (TS-BN-I for short) exhibited a high engineering strain up to 14% before fracture, almost one order of magnitude greater than those (around 1%) of hBN ceramics and other typical engineering ceramics (Fig. 2e and Supplementary Table 2). Accompanying the large axial strain was a large transverse expansion up to around 7% (Fig. 2f and Supplementary Video 1). The compressive strength reached 626 MPa, five to ten times that of the ceramic sintered from hBN nanosheets and other commercial hBN ceramics (Fig. 2a and Supplementary Table 3). With prolonged sintering time and higher sintering temperature, both deformability and compressive strength of TS-BN decreased; this was clearly related to growth of the ‘pure’ hBN phase (ideal AA′ stacking order and diminishing laminated structure of nanoplates) in the bulk. Indeed, for the ceramic sintered at 1,800 °C, with a complete transformation from oBN to hBN as indicated from XRD and SAED (Fig. 1a and Extended Data Fig. 2j), fracture strain and compressive strength were reduced drastically compared with those of TS-BN-I, with values similar to those of hBN ceramic.

Cyclic uniaxial compression tests were carried out on the TS-BN-I ceramic. Figure 2b and Supplementary Video 2 show the results of a single cyclic compression test, where axial compressive stress was gradually increased to 619 MPa (with a corresponding strain of 13%) and then released completely. No visible cracks occurred during the test, and the sample remained intact, with a remarkable residual plastic deformation of around 8%. In a multiple cyclic test (Fig. 2c and Supplementary Video 3), the stress–strain curves of one load–unload cycle formed a closed hysteresis loop with maximum stress below 300 MPa, showing remarkable anelastic behaviour. The hysteresis loop opened up under higher stress (Fig. 2c), indicating irreversible permanent plastic deformation.

The elastic–plastic deformability of the TS-BN-I ceramic stood out among those of all bulk polycrystalline ceramics (Fig. 2e and Supplementary Table 2). In the cyclic compressive test, the enclosed area of a hysteresis loop is a measure of the absorbed mechanical energy, indicating a high damping capacity^22^. The dissipated energy versus compressive stress plot is shown in Fig. 2d for the TS-BN ceramic. Its superiority over other ceramics was striking. In the anelastic deformation stage (the red zone in Fig. 2d), the dissipated energy increased linearly with stress, reaching around 1.0 MJ m^−^^3^ with a maximum stress of 300 MPa. This was one order of magnitude higher than that of polycrystalline graphite^23^ and slightly higher than that of Ti_3_SiC_2_ (ref. ^22^). With higher compressive stress (the purple zone in Fig. 2d), the dissipated energy increased abruptly to a maximum of 45 MJ m^−3^ with introduction of plastic deformation. This value was two orders of magnitude higher than that of commercial hBN ceramics (0.26 MJ m^−3^) and significantly higher than those of other engineering ceramics, such as SiC (15 MJ m^−^^3^) and Mg partially stabilized zirconia (28 MJ m^−^^3^)^24,25^. Therefore, the TS-BN ceramic is an excellent candidate as an impact absorber. The blue zone in Fig. 2d shows the dissipated energy estimated from a load-to-failure test (Fig. 2a), where an even larger value (as high as 70 MJ m^−^^3^) was achieved.

## Deformation mechanisms

**Fig. 3:**
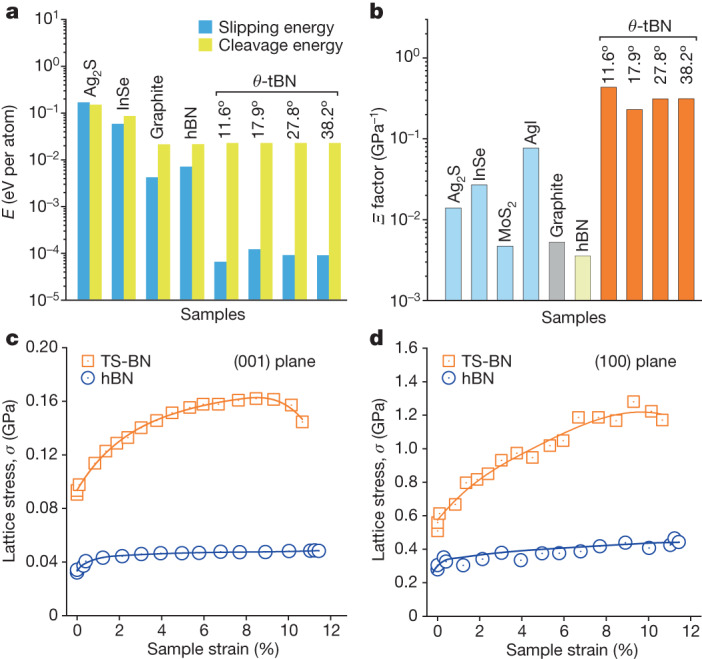
Origin of superhigh deformability and strength of TS-BN ceramic. **a**, Calculated slipping energy and cleavage energy of the hypothetical *θ*-tBN crystals. Compared with hBN, the introduction of twisted stacking into the structure significantly reduced slipping energy while maintaining cleavage energy. Other vdW layered materials are shown for comparison^18^. **b**, Intrinsic deformability factors (*Ξ*) of hypothetical *θ*-tBN crystals were two orders of magnitude higher than that of hBN and even higher than those of Ag_2_S and InSe, which are known to have ultrahigh room-temperature deformability^17,18^. **c**,**d**, Average differential stresses (that is, strengths) of the (001) (**c**) and (100) (**d**) lattice planes during triaxial compression tests (with mean stress of around 1.5 GPa) derived from in situ synchrotron radiation XRD observations. The strength of TS-BN (orange squares) was much higher than that of hBN (blue circles).

To examine the effect of twisted stacking in TS-BN on *Ξ* (ref. ^18^), we built several hypothetical crystals by twisting every other layer in hBN with an angle of *θ* (*θ*-tBN for short; Supplementary Fig. 4a). First-principles density functional theory (DFT) calculations indicated that twisting between adjacent BN layers could reduce *E*_s_ by two orders of magnitude, with little impact on *E*_c_ or *Y*. As a result, *Ξ* increased drastically compared with that of hBN (Fig. 3a,b and Supplementary Table 5). Owing to the very high density of twisted interfaces in the *θ*-tBN model, the calculated *Ξ* was even higher than those of Ag_2_S and InSe with ultrahigh room-temperature deformability^17,18^. In reality, TS-BN contains a lower density of twisted layers than *θ*-tBN. Nonetheless, twisted interfaces in laminated BN nanoplates have a critical role in boosting intrinsic deformability of BN ceramics.

**Fig. 4:**
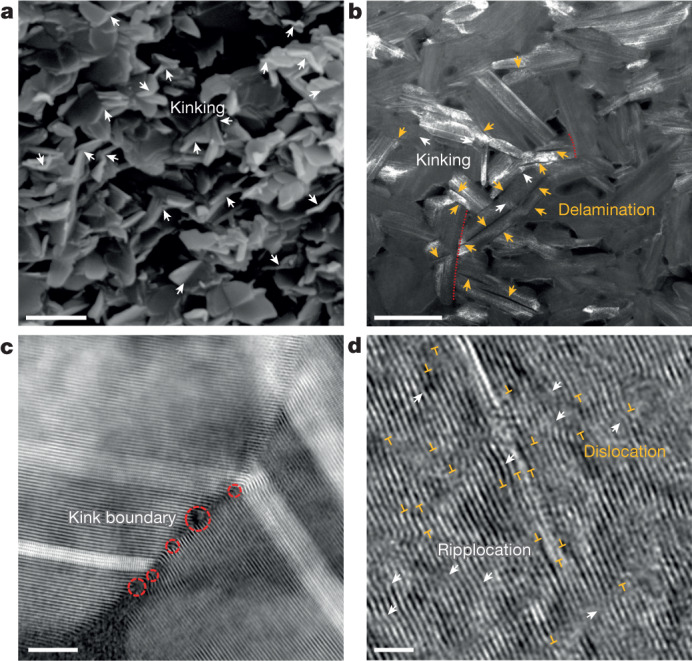
Deformation modes of TS-BN ceramic. **a**, Fractured surface showing numerous nanoplates that are sharply bent into two halves by kinks (white arrows). **b**, DF-STEM image showing kinking (white arrows) and delamination (orange arrows) of nanoplates in ceramic. The delaminating plane is the basal plane, through which nanoplates are ‘peeled’ into multiple plates. The red dotted line indicates a delamination that is blocked by the surrounding nanoplates with different orientations. **c**, HAADF-STEM image of a kink boundary with local defects (red circles). **d**, TEM image showing ripplocation (arrows) and dislocation (⊥) between basal atomic layers. Scale bars, 500 nm (**a**), 200 nm (**b**), 5 nm (**c**), 2 nm (**d**).

**Extended Data Fig. 6:**
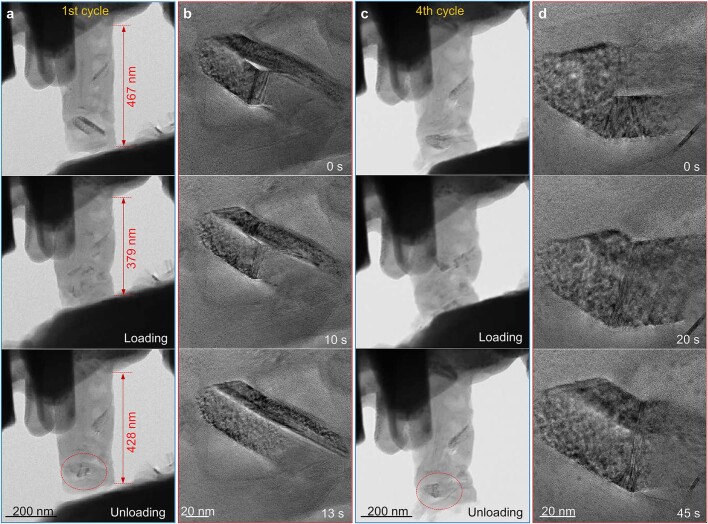
In-situ TEM compression test on a TS-BN nanopillar. This figure summarizes results of the first and fourth deformation cycles. **a** and **b**, The first loading-unloading cycle. **a**, Bright-field images showing length change in nanopillar during compression and decompression. After total release of stress, the sample recovers about half of the total length change defined by the difference between the initial length and the length under maximum deformation. **b**, Anelasticity behavior observed after unloading, showing a gradual disappearance of deformations including kink bands and delaminations in the nanoplate. During the recovery process, the surrounding nanoplates appear to move around to accommodate the shape change in the plate, leaving no voids. **c**, The fourth loading-unloading cycle causing severe plastic deformation in ceramic nanopillar. **d**, Hysteresis deformation after the fourth cycle, showing that the kinking deformation moved and eventually remained in the nanoplate. **b** and **d** were the enlarged areas of the red dotted ellipses in **a** and **c** respectively.

We further explored deformation mechanisms by investigating the microstructure of TS-BN ceramics after compressive fracture. Figure 4a shows the typical morphology of a fractured surface, in which numerous kinked nanoplates are present owing to severe compression (marked by white arrows). Dark-field (DF)-STEM observations showed abundant nanoplate kinking (white arrows in Fig. 4a,b) and delamination in the nanoplates (orange arrows in Fig. 4b). The observed microstructure indicates that delamination is localized to within individual nanoplates and cannot propagate across adjacent nanoplates to form microcracks. Thus, the three-dimensional bulk TS-BN is a nanoarchitectured material characterized by interlocked nanoplates. Its microstructure contrasts sharply with that of compressed hBN ceramics, where microcracks propagate readily along the basal planes of nanolaminae (Supplementary Fig. 2), resulting in low deformability. In the deformed TS-BN sample, defects are primarily localized within kink boundaries (Fig. 4c), with large numbers of ripplocations and dislocations observed in the nanoplates (Fig. 4d), all of which are absent from the initially ordered TS-BN nanoplates (Fig. 1d). These defects—that is, kinking, delamination, ripplocation and dislocation—contribute to the plastic deformation in TS-BN ceramics. In situ room-temperature uniaxial compression tests on TS-BN-I nanopillars by TEM showed deformability due to kinking and delamination (Extended Data Fig. 6). This extrinsic deformation mechanism is similar to that in layered MAX phase ceramics under compression^26,27^ but different from those in micro- and nanoscaled samples owing to dislocation slip^11–13^ and phase transformation^14,15^.

We also conducted triaxial deformation using in situ synchrotron radiation XRD at room temperature^28^. A bulk TS-BN-I sample was loaded quasi-hydrostatically to about 1.5 GPa before an axial strain was applied along the cylindrical axis (see Methods for experimental details). Differential stresses were estimated on the basis of lattice strains of the (002) and (100) planes by assuming that the elastic constants of these planes could be approximated by those of hBN under the corresponding pressure (Fig. 3c,d). The differential stress values thus obtained were highly anisotropic, with maximum stress normal to the (100) planes (up to 1.2 GPa) nearly eight times that normal to the (001) planes (0.16 GPa), which are dominated by vdW interactions. For comparison purposes, an hBN sample with identical dimensions was also tested. As pressure suppresses brittle behaviour, hBN can be deformed plastically up to around 11.5% axial strain, with differential stresses on the (100) and (001) planes of about 0.4 and 0.05 GPa, respectively; both of these values are about one-third of their TS-BN-I counterparts. TS-BN-I exhibited significant strain hardening, whereas hBN showed only slight strain hardening. We attribute the exceptional mechanical properties of TS-BN to the intrinsically easy interlayer slipping that significantly reduces local stress concentration, as well as the three-dimensional interlocked nanoarchitecture that provides extrinsic constraints by confining brittle deformation through kinking, delamination and ripplocation within individual nanoplates. As a result, greater deformability, load-bearing capacity and strength have been achieved in the TS-BN ceramic.

**Extended Data Fig. 7:**
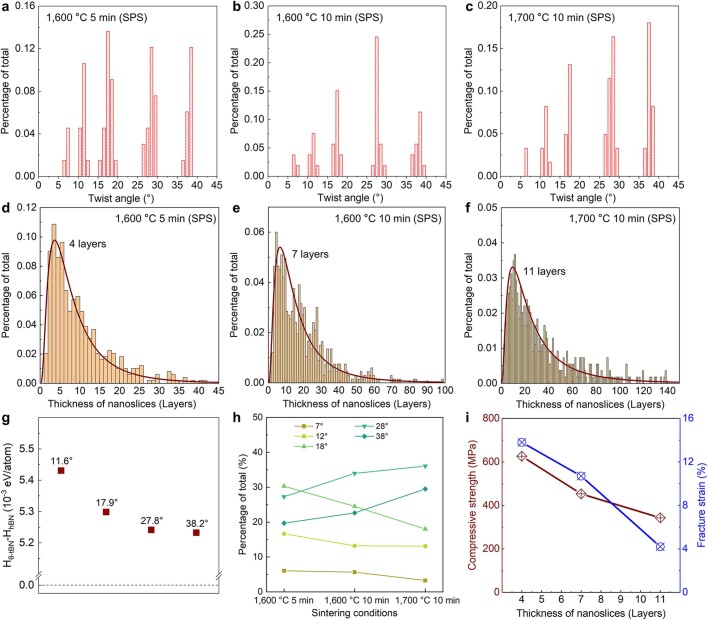
Statistical distribution of twist angles and nanoslice thickness, and the relationships between synthesis parameters, microstructure, and mechanical properties in SPS sintered TS-BN ceramics. **a**-**c**, Distribution of twist angles in ceramics obtained at various conditions, 1,600 °C, 5 min (**a**); 1,600 °C, 10 min (**b**); and 1,700 °C, 10 min (**c**). The twist angles are concentrated at four main angles (~38°, ~28°, ~18°, and ~12°) and a non-dominant angle of ~7°. Supplementary Fig. 4a shows the hypothetical twist-stacked crystal structures with twist angles of 38.2°, 27.8°, 17.9°, 11.6°, and 7°, respectively. **d**-**f**, Thickness of twistedly stacked nanoslices in ceramic nanoplates, 1,600 °C, 5 min (**d**); 1,600 °C, 10 min (**e**); and 1,700 °C, 10 min (**f**). As the sintering temperature and time increase, the average thickness of nanoslices increases from 4 layers to 11 layers, leading to a decrease in the density of twisted interface. **g**, DFT calculated enthalpies of hypothetical twisted-layer BN structures with respect to hBN. **h**, Percentage variation of twist angles with sintering temperature and holding time. **i**, Relationships between thicknesses of nanoslices and the compressive strength (and fracture strain) of TS-BN ceramics.

**Extended Data Fig. 8:**
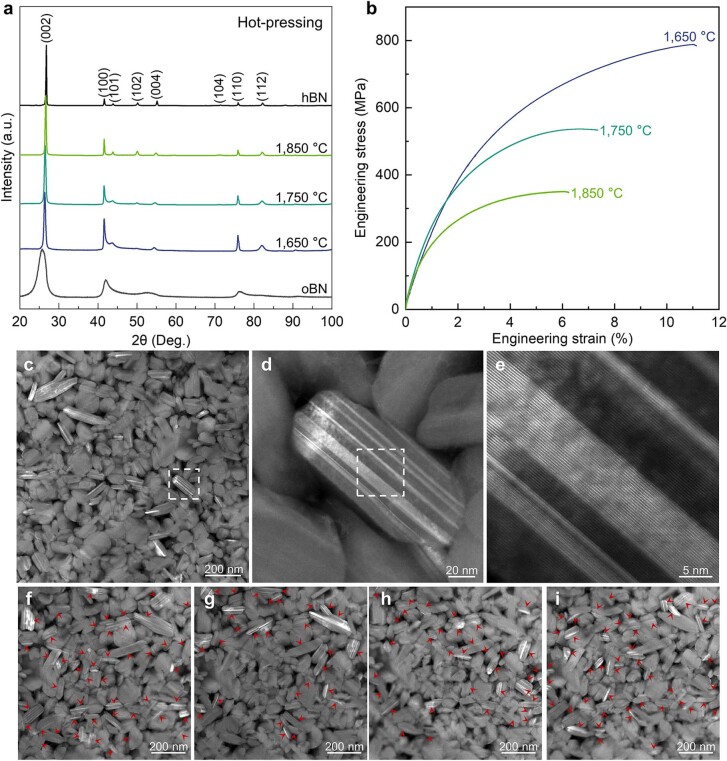
Structure and properties of TS-BN ceramics prepared by hot-pressing sintering. **a**, XRD patterns of TS-BN ceramics prepared at various conditions (50 MPa and 1,650 − 1,850 °C, 5 min), showing similar diffraction results as those of SPS sintered ceramics (Fig. 1a). The crystal planes of hBN are marked in the figure. Compared with hBN, the introduction of twist-stacked structure results in the left shift of the (002) diffraction peak, indicating an increase in interlayer spacing. Moreover, the additional diffraction peaks between (100) and (004) diffraction lines of hBN appear in twist-stacked structures (Extended Data Fig. 5), causing the high background intensity observed in the diffraction patterns. **b**, Engineering stress−strain curves during uniaxial compression. The TS-BN samples have a compressive strength up to 773 MPa and fracture strain of 10.2%, demonstrating the reproducibility of large room-temperature deformability and high strength in hot-pressed sintered bulk ceramics. The slight differences in strength and strain of hot-pressing (or SPS) sintered samples are due to the differences in the microstructure (e.g. twist angle and nanoslice thickness) of the samples. **c**-**e**, Gradually enlarged HAADF-STEM images, indicating that the bands with different contrasts in the nanoplate correspond to the laminated nanoslices with different in-plane orientations. **f**-**i**, HAADF-STEM images collected from the same region at different α-, β-tilt angles: **f** (10.4°, −10.7°), **g** (−1.1°, 15.5°), **h** (−20.2°, 6.8°), and **i** (−10°, −10°). Red arrows show the appearance of nanoplates with bright and dark bands at different tilt angles in STEM, indicating the prevalence of twisted stacking structures in the ceramic nanoplates.

**Extended Data Fig. 9:**
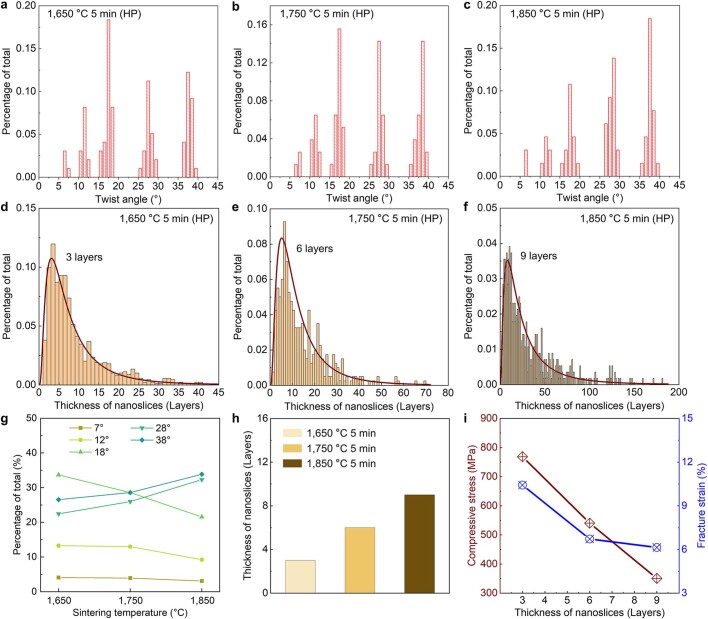
Statistical distribution of twist angles and nanoslice thickness, and the relationships between synthesis parameters, microstructure, and mechanical properties in hot-pressing (HP) sintered TS-BN ceramics. **a**-**c**, Distribution of twist angles in the ceramics sintered at 1,650 °C (**a**), 1,750 °C (**b**), and 1,850 °C (**c**). The distribution of twist angle is similar as that of SPS sintered samples, concentrated around 38°, 28°, 18°, 12°, and 7°. **d**-**f**, Thickness of nanoslices in ceramics sintered at 1,650 °C (**d**), 1,750 °C (**e**), and 1,850 °C (**f**). **g**, Percentage variation of twist angles with sintering temperature. As the sintering temperature increases, the proportion of 27.8° and 38.2° gradually increased. **h**, Average thickness of nanoslices in ceramics sintered at different temperature. As the sintering temperature increases, the average thickness of nanoslices increases from 3 layers to 9 layers, which is slightly less than that of SPS sintered ceramics. **i**, Relationships between the nanoslice thickness and the strength/strain of TS-BN ceramics. With the nanoslice thickness increases, the strength and fracture strain of ceramics gradually decrease. The distribution of twist angles, the percentage change of twist angles, and the variation of nanoslice thickness with sintering temperature, as well as the relationship between nanoslice thickness and the strength/strain of hot-pressing sintered ceramics, are similar to the situation in SPS sintered ceramics. This shows that pressure, temperature, and holding time are the key factors controlling the microstructure and properties of TS-BN ceramics; effects of electronic current applied in SPS synthesis are negligible.

**Extended Data Fig. 10:**
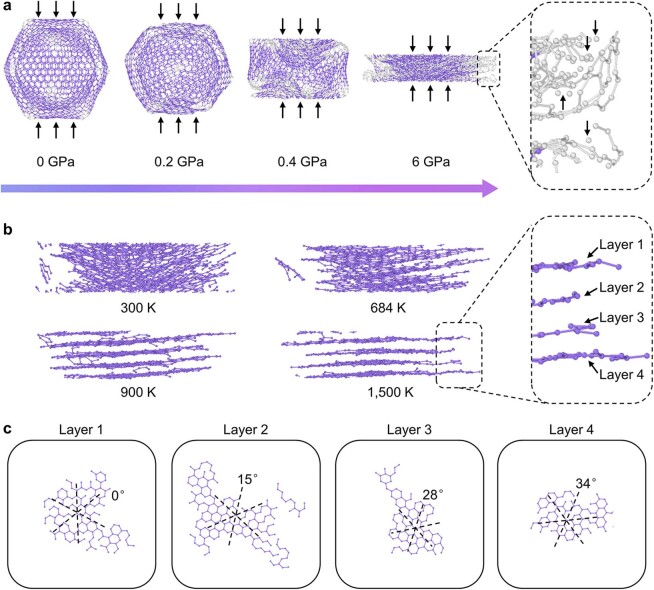
Phase transition from onion-like BN to twisted-layer structure during the compression and heating. **a**, Evolution of a two-shell onion-like BN structure during compression from 0 GPa to 6 GPa at 300 K. **b**, Structural evolution at 6 GPa during heating from 300 K to 1,500 K. In both **a** and **b**, the violet colored part represents graphene-like structure. The grey part represents an unidentified structure. Atoms and bonds are colored according to the polyhedral template matching algorithm. Arrows in the enlarged image mark unbonded atoms. The cutoff value for the bond is 1.52 Å. With the increase of temperature, the collapsed onion-like BN structure transformed into a layered structure. **c**, Top view of each layer formed at 6 GPa, 1,500 K. Twist angles of other layers relative to the layer 1 are shown. The results indicate that the onion-like structure underwent a series of changes under compression and heating, including distortion, collapse, fragmentation, and subsequent recrystallization, leading to the formation of the twisted-layer structure. Note that the size of onion-like BN structure in the theoretical simulation is significantly smaller than that used in the experiment. As a result, the pressure required for simulation is higher than the actual condition.

We also established relationships between SPS sintering parameters, microstructures (twist angle distribution, twisted nanoslice thickness) and mechanical properties (Extended Data Fig. 7). Statistically, the measured twist angles were mostly concentrated around 38°, 28°, 18°, 12° and occasionally around 7° (Extended Data Fig. 7a–c). Some angles were also observed in moiré patterns (Fig. 1e and Extended Data Fig. 4). With increasing sintering temperature and holding time, percentages of the 7°, 12° and 18° twist angles decreased, whereas percentages of the 28° and 38° twist angles increased owing to the favourable thermodynamic stability of the structure (Extended Data Fig. 7g,h). The introduction of twisting between adjacent BN layers increases the deformability factor by two orders of magnitude, but the deformability factor varies slightly with twist angles (Fig. 3b and Supplementary Table 5). With increasing sintering temperature and holding time, average thickness of twist-stacked nanoslices in nanoplates increases from 4 to 11 layers (Extended Data Fig. 7d–f). The increase in nanoslice thickness means an increase in the size of twisted-stacking units and a decrease in twisted-layer interfaces, resulting in a decrease in the strength and deformability of TS-BN ceramics (Extended Data Fig. 7i). Finally, we have successfully reproduced the twisted-layer BN ceramics using hot-pressing sintering under conditions similar to the SPS synthesis (Extended Data Figs. 8 and 9 and Supplementary Table 4). The atomistic mechanism of transition from oBN to twisted-layer BN under pressure and temperature was investigated using molecular dynamics simulations (Extended Data Fig. 10). The ability to hot-pressing sinter TS-BN ceramics means that the materials can be much more easily scaled up for practical applications.

By introducing a twisted laminated structure to the nanoplates and building a three-dimensional interlocked nanoarchitecture, we have achieved high deformability, plasticity and strength of a vdW BN ceramic. The toughness and strength of the twisted-layer ceramic are expected to be further improved by the addition of BN or carbon nanofibres or nanotubes, as well as by adding a second ceramic phase. The realization of plastic deformation shows that ceramics can be truly permanently deformed, similar to metals, without fracture. The structural architecture strategy demonstrated in this study also sheds light on the development of other layered vdW engineering ceramics with simultaneously enhanced room-temperature deformability, strength, toughness and energy absorption.

## Methods

### Sample preparation

The oBN nanoprecursors were prepared by the chemical vapour deposition method^16,20,29^; the raw materials used in the current work were trimethyl borate and ammonia. The oBN particle size ranged from 50 to 500 nm, with an average size of around 180 nm (Supplementary Fig. 1). A DR.SINTER SPS system and HIGH MULTI 10000 hot-pressing sintering device were used to sinter the precursors, respectively. For SPS sintering, a pressure of 50 MPa was applied first, followed by rapid heating to the target temperature at a rate 100 °C per min. Temperature was monitored with an on-line infrared thermometer during sintering. After 5–10 min at the target temperature, the power was cut off, and the pressure was released. The as-sintered specimens were left in the SPS until they had cooled to room temperature; then, they were taken out and polished. TS-BN ceramics can be synthesized between 1,600 and 1,800 °C by SPS. The ceramic synthesized at 1,500 °C was a composite consisting of TS-BN and residual untransformed oBN (Supplementary Fig. 5). For hot-pressing sintering, the same sintering pressure of 50 MPa was applied first, followed by gradual heating to the target temperature at a rate of 10 °C per min. The holding time was set to 5 min, and then the heating was stopped, and the pressure was released. Densities of the as-sintered specimens were measured according to the Archimedes principle.

### XRD and Raman spectroscopy

XRD was used to characterize both the oBN nanoprecursors and the as-sintered BN ceramics, using a Rigaku diffractometer (SmartLab, Rigaku) with Cu Kα radiation (*λ* = 0.15418 nm). The applied voltage and current were 40 kV and 40 mA, respectively, with a step size of 0.02° at a scanning rate of 1° per min. Raman spectra were also collected at room temperature using a Horiba Jobin Yvon LabRAM system with a laser wavelength of 473 nm. The size of the laser spot was approximately 1 μm.

### TEM sample preparation

oBN nanoparticles were dispersed in ethanol solution by ultrasonic treatment, drop-casted on to a carbon-coated copper grid and then dried before TEM observation. Sintered BN ceramics were first crushed and ground in an agate mortar; then, small nanoplates from ceramics were used to prepare TEM samples in the same way as above for oBN. In addition, thin foils were cut from as-sintered bulk samples for TEM observation using a focused ion beam (Helios 5 CX DualBeam, ThermoFisher). The foils were further milled to less than 100 nm and polished by Ar-ion milling (NanoMill; Model 1040, Fischione) to remove surface damage.

### Microstructure characterization

We used scanning electron microscopy (Verios, ThermoFisher) to characterize the oBN nanoparticles and fracture morphology of BN ceramics. More detailed microstructure was characterized with a scanning transmission electron microscope (Talos F200X, ThermoFisher) operated at an accelerating voltage of 200 kV and a spherical-aberration-corrected scanning transmission electron microscope (Themis Z, ThermoFisher) operated at an accelerating voltage of 300 kV. HAADF images were collected by combining 20 frames from acquired series with drift correction (DCFI in Velox software, Thermo Fisher). The probe convergence angle was set to 25 mrad, and the collecting angle for HAADF was set to 65–200 mrad to eliminate the coherent scattering effect.

### First-principles calculations

We constructed twist-layer BN structures using the Materials Visualizer module of the Materials Studio software^30^. Calculations were performed on the basis of DFT as implemented in the CASTEP code^31^. Ultrasoft pseudopotentials were used^32,33^. We used the local density approximation exchange-correlation functional of Ceperley and Alder parameterized by Perdew and Zunger^34,35^ to perform structural optimization and calculations of total energies and elastic properties. A *k*-point sampling^36^ of 0.04 × 2π Å^−1^ and a plane-wave cutoff of 570 eV were applied. The Broyden–Fletcher–Goldfarb–Shanno^37^ minimization scheme was used for geometry optimization. Structural relaxation was stopped when the total energy changes, maximum ionic displacement, stress and ionic Hellmann–Feynman force were less than 5.0 × 10^−6^ eV per atom, 5.0 × 10^−4^ Å, 0.02 GPa and 0.01 eV Å^−1^, respectively. The elastic moduli of the investigated structures were calculated in the linear elastic strain range. Selected calculation parameters were tested to ensure that energy convergence was less than 1 meV per atom. To validate our computational scheme, benchmark calculations were conducted for the hBN structure. The calculated lattice parameters of *a* = 2.49 Å and *c* = 6.48 Å were in good agreement with experimental values of *a* = 2.50 Å and *c* = 6.66 Å (ref. ^38^). The calculated bulk modulus (27.9 GPa) of hBN was in agreement with the experimental value (25.6 GPa)^39^.

### Deformability factor calculation

The ability of layered vdW materials to deform without fracture can be characterized by the deformability factor *Ξ* = (*E*_c_/*E*_s_)(1/*Y*) (in units of GPa^−1^)^18^, where *E*_c_ and *E*_s_ are the cleavage energy and slipping energy, respectively, and *Y* is the in-plane Young’s modulus along the slip direction. The *E*_c_/*E*_s_ ratio quantifies the plasticity of the material that conforms to the criterion proposed by Rice et al.^40,41^. Interlayer interactions and the relative glide of the twist-stacked BN structures were simulated on the basis of DFT calculations. The slipping step was kept at 0.3 Å during the simulation. For each step, the energy of the most stable configuration was obtained by geometrically optimizing only the interlayer distance. The (001) plane uniformly slipped along the [210] direction, which is considered to be the lowest-energy sliding direction in hBN^42^. We obtained the energy as a function of the slip distance over the range of periodic distances. The energy difference between the slip distance at maximum energy (*E*_max_) and no slip (*E*_0_) was used to represent the energy barrier to overcome resistance to slip, that is, *E*_s_ = *E*_max_ − *E*_0_ (ref. ^18^). The energy difference between the infinite interlayer distance (*E*_inf_) and *E*_max_ was considered to represent the cleavage energy, *E*_c_ = *E*_inf_ − *E*_max_ (ref. ^18^). An interlayer distance of 10 Å was used to calculate *E*_inf_, which safely ensured that there was no interlayer interaction.

### Molecular dynamics simulation

The phase transition process from oBN to a twisted-layer structure was simulated by molecular dynamics with the large-scale atomic/molecular massively parallel simulator code^43^. An extended Tersoff potential was chosen to describe the interatomic interaction^44^; this has been widely used to investigate the microstructural evolution of hBN^45,46^. In this work, a 10 × 10 × 4 nm^3^ supercell containing a two-shell BN nano-onion at the centre was constructed. The two-shell BN nano-onion structure was constructed using the method reported in our previous work^21^. The outer (inner) shell corresponded to B_750_N_750_ (B_460_N_460_), and the diameter of the BN onion was 3.61 nm. Periodic boundary conditions and isothermal–isobaric (NPT) ensemble were applied in the simulations. Each supercell was first optimized with the conjugate gradient algorithm and then relaxed for 20 ps at room temperature. Following the relaxation, the supercell was compressed uniaxially along the *z* direction to a given pressure (6 GPa) within 200 ps and finally heated to the target temperature (1,500 K) within 2 ns. Atomic configurations were visualized and analysed with the help of the Open Visualization Tool package^47^. The local structural environment of the atoms was identified using the polyhedral template matching algorithm^48^.

### Mechanical property characterization

Uniaxial compression tests were performed in the MTI mechanical property testing system (MTII/Fullman SEMtester 2000) at room temperature with a strain rate of 1 × 10^−4^ s^−1^. Ceramic specimens were machined into cylinders with diameter of 2.7 mm and length of 4.0 mm (length to diameter ratio: approximately 1.5). Both ends of the cylinders were polished with diamond powder of around 0.5 μm. Parallelism between the two ends was within 0.01 mm. A thin copper foil was placed between the sample and the tester to reduce stress concentration on the contact area. Thin nickel (Ni) film was deposited on the sample surface to form markers. Each compression process was recorded in situ with a digital video recorder. Sample strain was estimated by measuring the change in distances between Ni markers. We conducted at least five tests for uniaxial compressive properties on bulk samples obtained from SPS and hot-pressing sintering.

Tensile tests were carried out at a strain rate of 1 × 10^−4^ s^−1^. The specimens were processed into I-shape with an effective tensile length of 6 mm and a rectangular cross section of 1 mm × 1.5 mm. Flexural strength was measured using the three-point bending method. The specimens were processed into cuboids with a size of 1 mm × 2 mm × 12 mm. The span length was 11 mm, and the loading rate of the indenter was set to 0.1 mm min^−1^. Flexural strength (*σ*_f_) was calculated as follows:

1 $${{\sigma }}_{{\rm{f}}}=\frac{3FL}{{2bh}^{2}},$$

where *F* is the maximum load until the specimen fractured, *L* is the span length, and *b* and *h* are the width and height of the specimen, respectively.

Fracture toughness of the specimens was determined using the single-edge V-notched beam method. The specimens were processed into cuboids with a size of 1 mm × 1.5 mm × 5 mm. A straight-through V-notch with a depth of approximately 0.5 mm was cut in the specimen using a femtosecond laser (Astrella-1K-USP). The radius of the notch tip was less than 10 μm, the span length was 4 mm and the crosshead loading rate was 0.1 mm min^−1^. Fracture toughness (*K*_IC_) was calculated using the following equations:

2 $${K}_{{\rm{I}}{\rm{C}}}=\left(\frac{FL}{B{W}^{1.5}}\right)\left\{1.5{\left(\frac{a}{W}\right)}^{0.5}Y(\frac{a}{W})\right\},$$

3 $$Y\left(\frac{a}{W}\right)=1.964-2.837\left(\frac{a}{W}\right)+13.711{\left(\frac{a}{W}\right)}^{2}-23.25{\left(\frac{a}{W}\right)}^{3}+24.129{\left(\frac{a}{W}\right)}^{4},$$

where *F* is the maximum load until the specimen fractured, *L* is the span length, *B* is the specimen width, *W* is the specimen height and *a* is the notch depth. Tests for tensile strength, flexural strength, Young’s modulus and fracture toughness were repeated at least five times.

### In situ synchrotron radiation XRD measurements

In situ triaxial compression tests were performed using a deformation-DIA apparatus coupled with synchrotron X-rays, at the GSECARS 13-BM-D beamline of the Advanced Photon Source at the Argonne National Laboratory, USA. Details of the deformation-DIA module and the sample assembly can be found elsewhere^28,49^. The specimens were cylinders of 2.5 mm in diameter and 3.5 mm in length. The specimen was first compressed to a hydrostatic pressure of around 1.5 GPa; then, the differential rams were advanced to shorten the sample at a constant speed (strain rate = 1 × 10^−5^ s^−1^) at room temperature.

The incident monochromatic beam (45 keV) was collimated to tungsten carbide (WC) slits of 200 mm × 200 mm. Detector tilt and rotation relative to the incident beam were calibrated with a CeO_2_ standard using the Dioptas program^50^. XRD patterns and radiographs of the sample were collected automatically during deformation. Exposure times for XRD and radiographs were 300 s and 10 s, respectively.

The strain of specimen was defined as *ε* = (*l*−*l*_ε_)/*l*, where *l* and *l*_ε_ are the sample lengths at the initial state and under compression, respectively. Lattice strain of the (002) and (100) reflections was defined as

4 $$\frac{{d}_{{\rm{hkl}}}(\varphi ){-d}_{{\rm{P}}({\rm{hkl}})}}{{d}_{{\rm{P}}({\rm{hkl}})}}{=Q}_{{\rm{hkl}}}({1-{\rm{3cos}}}^{2}\varphi ),$$

where *d*_P(hkl)_ is the hydrostatic pressure d-spacing, for which the right-hand side of equation (4) is zero. For each plane (hkl), *d*_(hkl)_ and *φ* were measured from the two-dimensional diffraction pattern (with cos*φ* = cos*θ*cos*δ*), *δ* being the true azimuth angle. *Q*_(hkl)_ and *d*_P(hkl)_ were extracted by fitting *d*_(hkl)_ versus *φ* according to equation (4). Differential stresses *σ*_(hkl)_ were calculated for planes from lattice strains *Q*_(hkl)_ according to:

5 $${\Delta \sigma }_{{\rm{hkl}}}{=6Q}_{{\rm{hkl}}}{G}_{{\rm{hkl}}},$$

where the ‘effective moduli’ *G*_hkl_ were calculated with elastic compliances *S*_ij_ from inversion of the stiffness tensor (*C*_ij_) for hBN. In the current work, elastic constants of *c*_33_ = 27 GPa and *c*_11_ = 811 GPa (ref. ^39^) were used to calculate *σ*_(002)_ and *σ*_(100)_ of layered BN structures, respectively, without considering pressure effects on these moduli.

### In situ TEM nanopillar compression

Nanopillars with diameter of around 200 nm and aspect ratio of around 2:1 from the sintered bulk ceramic were fabricated using a Ga ion beam at a voltage of 30 kV in a FEI Helios focused ion beam instrument. Initially, the samples were processed into pillars with a cross-sectional width of approximately 5 μm using relatively large currents from 21 nA to 7 nA. Subsequently, the pillars were milled to cylinders with diameter of 1 μm using low currents from 5 nA to 1 nA. Finally, the pillars were polished to the desired size of approximately 200 nm using small currents from 500 pA to 7.7 pA to minimize the damage layer.

In situ uniaxial compression tests were performed with a Hysitron Picoindenter instrument inside a transmission electron microscope (FEI Titan ETEM G2) operated with an accelerating voltage of 300 kV. The Hysitron PI-95 holder was equipped with a diamond punch joined to a MEMS transducer. In situ compression experiments were carried out in a displacement control mode, which has been proved to be more sensitive to transient phenomena. The displacement rate was kept at 10 nm s^−1^ during compression, corresponding to a strain rate of 1 × 10^−2^ s^−1^. The whole process was recorded using a digital video recorder for observation of the evolution of the microstructure.

## Online content

Any methods, additional references, Nature Portfolio reporting summaries, source data, extended data, supplementary information, acknowledgements, peer review information; details of author contributions and competing interests; and statements of data and code availability are available at 10.1038/s41586-024-07036-5.

### Supplementary information

Supplementary Information Supplementary Figs. 1–5 and Tables 1–5.

Supplementary Video 1 Single compression until fracture.

Supplementary Video 2 Single cyclic compression test.

Supplementary Video 3 Multiple cyclic compression test.

## Data Availability

The data that support the findings of this study are available within this article and its Supplementary Information. Further data are available from the corresponding authors upon request.
