# Supplementary material for: Twisted-layer boron nitride ceramic with high deformability and strength
Source: Nature. 2024 Feb 21;626(8000):779–84. doi: 10.1038/s41586-024-07036-5 (PMC10881384; doi:10.1038/s41586-024-07036-5)
Supplement: Supplementary file 1 — Supplementary Figs. 1–5 and Tables 1–5. [file 41586_2024_7036_MOESM1_ESM.pdf]

nature portfolio

https://doi.org/10.1038/s41586-024-07036-5

Supplementary information

Twisted-layer boron nitride ceramic with high deformability and strength

In the format provided by the authors and unedited

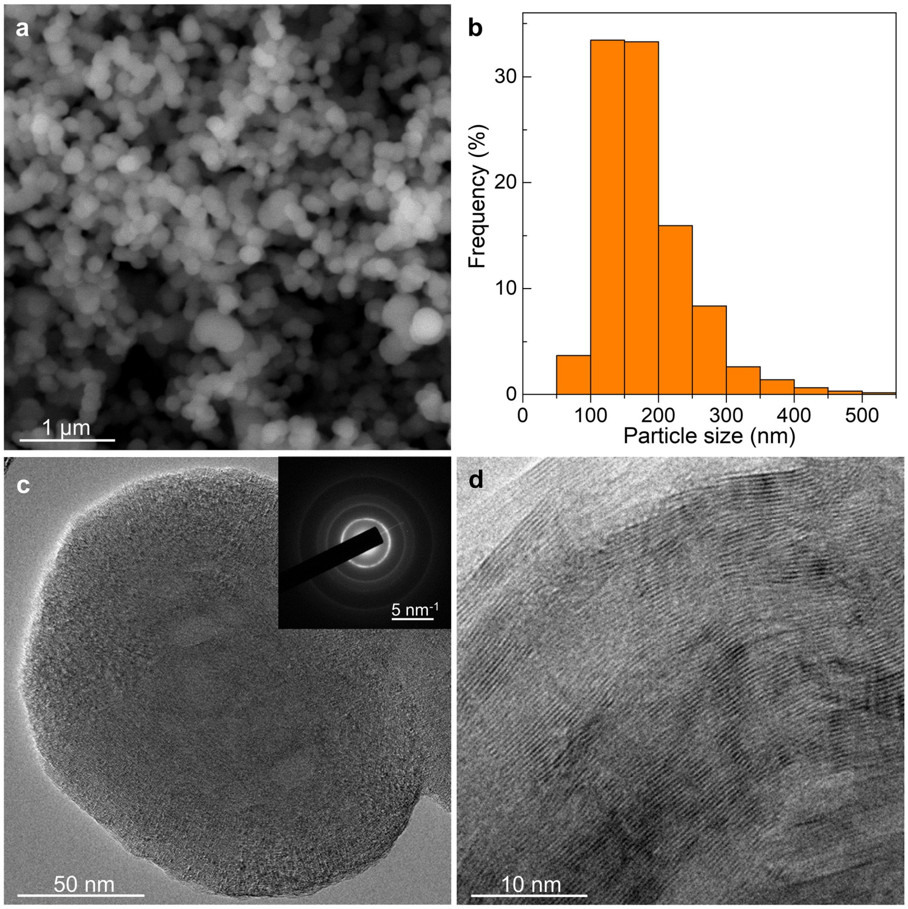

Supplementary Figure 1 | Microstructure of oBN nanoprecursor. a and **b,** SEM image and size distribution of oBN nanoparticles. The average size is ~180 nm. **c** and **d,** TEM images showing that oBN is composed of turbostratic spherical shells with abundant puckering and stacking faults. The inset in **c** is the corresponding SAED pattern.

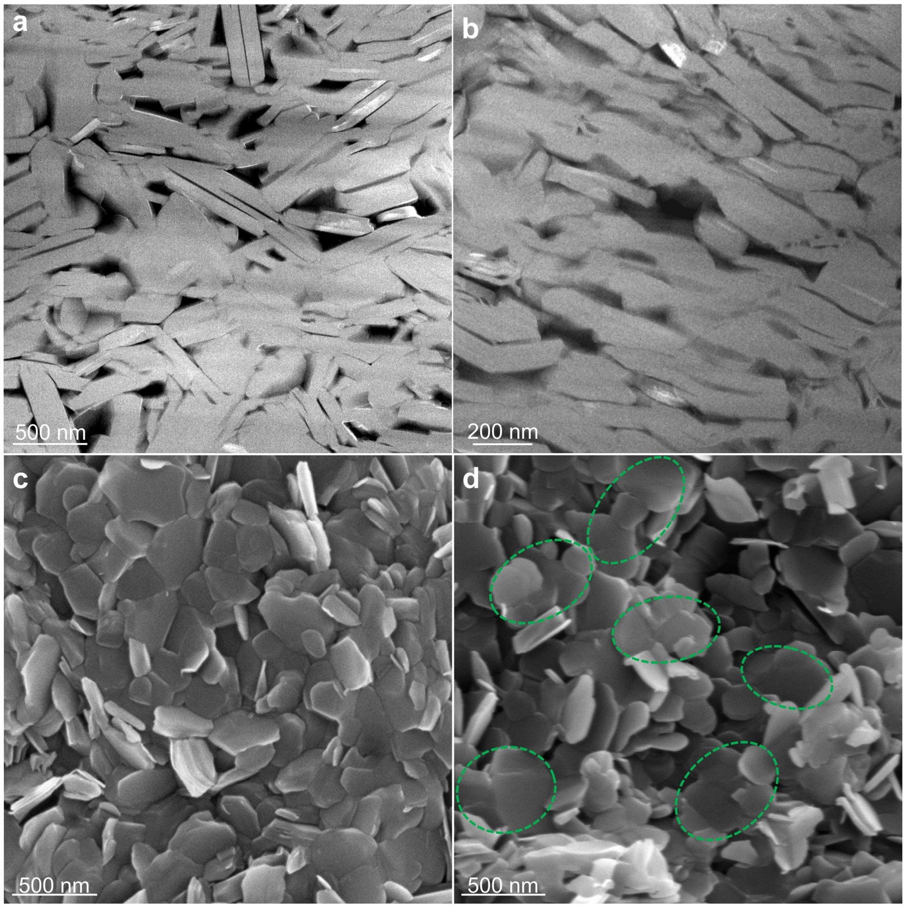

Supplementary Figure 2 | Microstructure of hBN ceramics prepared by SPS sintering hBN nanosheets before and after compression test. a, HAADF-STEM image before compression test. The BN nanoplates in ceramic have preferential orientation on the micrometer scale. **b**, HAADF-STEM image after compression test. Microcracks have propagated continuously along the basal planes of the preferentially oriented nanoplates. **c**, Fracture morphology formed by direct breaking hBN ceramic with mechanical pliers. This is the fresh sample without compression test. **d**, Fractured surface after a compression test. The micro-scaled cleavage surfaces composed of several oriented nanoplates were observed (green dotted areas), indicating the low compressive strength (135 MPa) and strain (2.1%) of hBN ceramics.

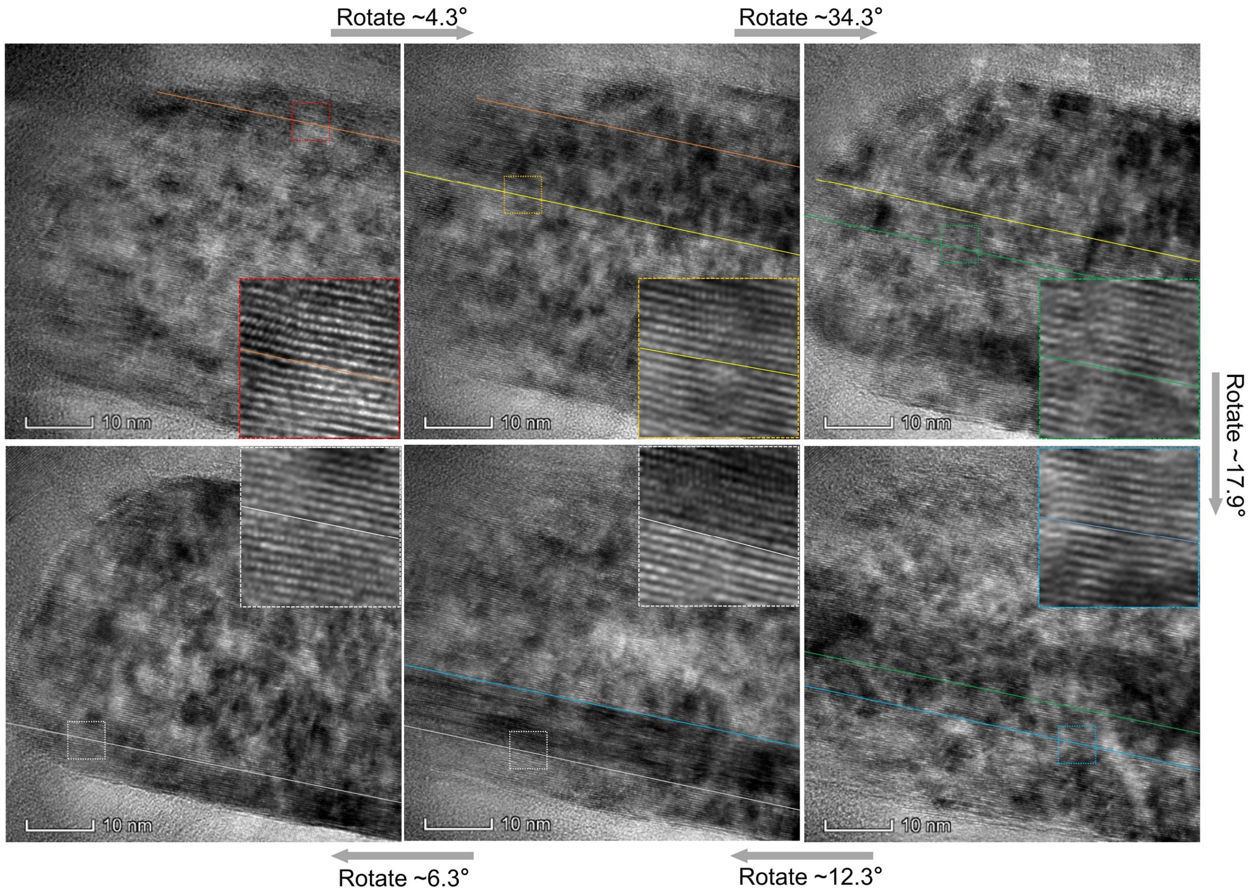

Supplementary Figure 3 | High-resolution transmission electron microscopy (HRTEM) images of a nanoplate with basal planes viewed edge-on. The sample is rotated with respect to the normal of the basal plane. Insets show an atom-resolution lattice region with a thickness of several to dozens of layers. As the sample is rotated, different regions of lattice fringes move in and out of focus, as indicated by the colored lines. These regions, referred to as nanoslices, have different orientational relations, but share the same basal plane normal, indicating that they are twisted relative to one another. Insets show the interfaces between adjacent nanoslices, and the atomic-resolution lattice and striped regions were observed at the interface boundary due to the different orientations.

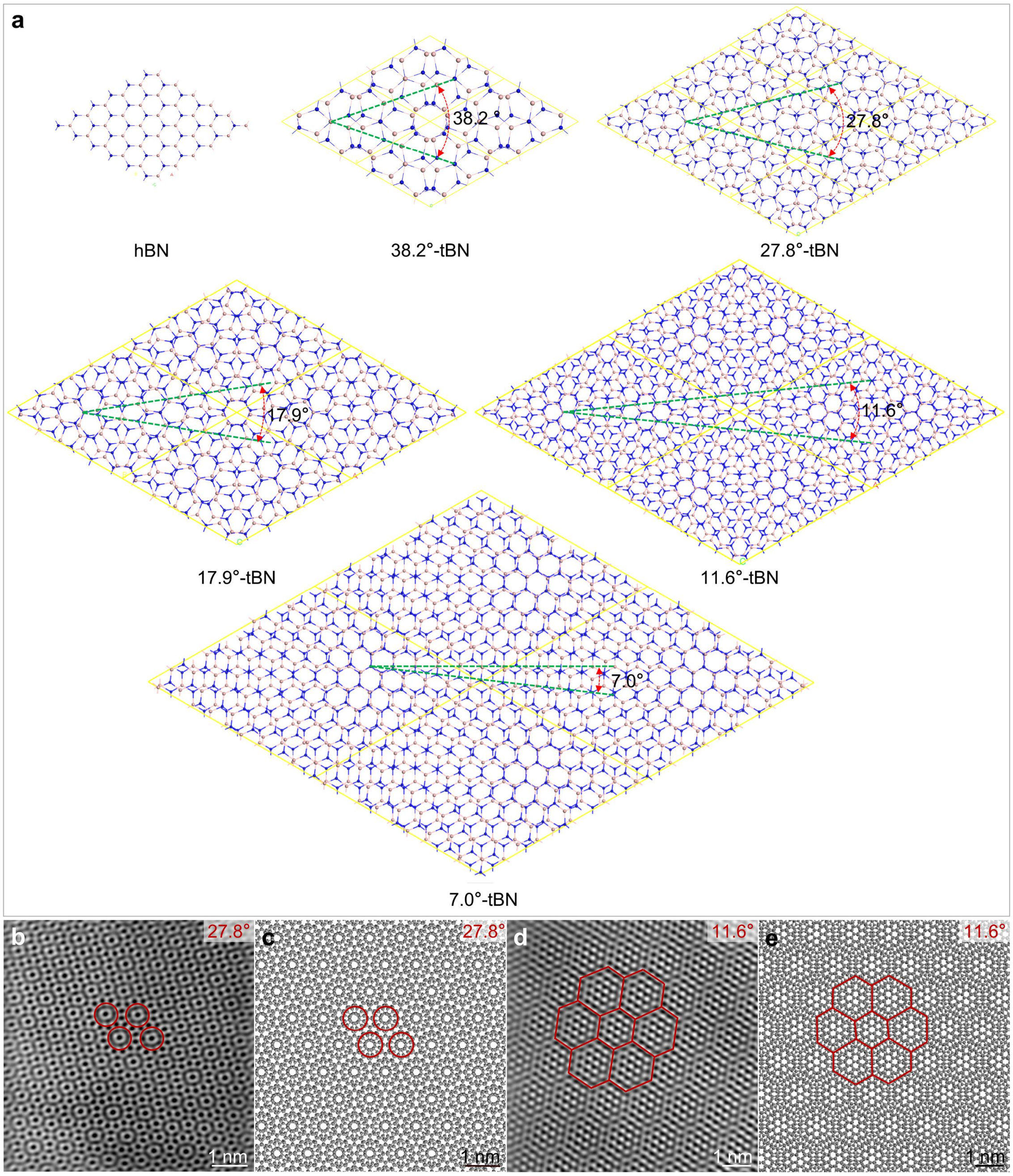

Supplementary Figure 4 | Hypothetical twist-stacked crystals constructed by twisting every other layer by an angle of θ in hBN (θ-tBN for short). a, Crystal structures of hBN and θ-tBN with twist angles of 38.2°, 27.8°, 17.9°, 11.6°, and 7.0°, respectively. **b** and **d,** Observed Moiré patterns in as-sintered TS-BN samples, in which the superimposed nanoslices have twist angles of 27.8° and 11.6°, respectively (Fig. 1e, Extended Data Fig. 4). **c** and **e**, Simulated moiré patterns of 27.8°-tBN and 11.6°-tBN structures. The simulated moiré patterns are in good agreement with the experimental observations.

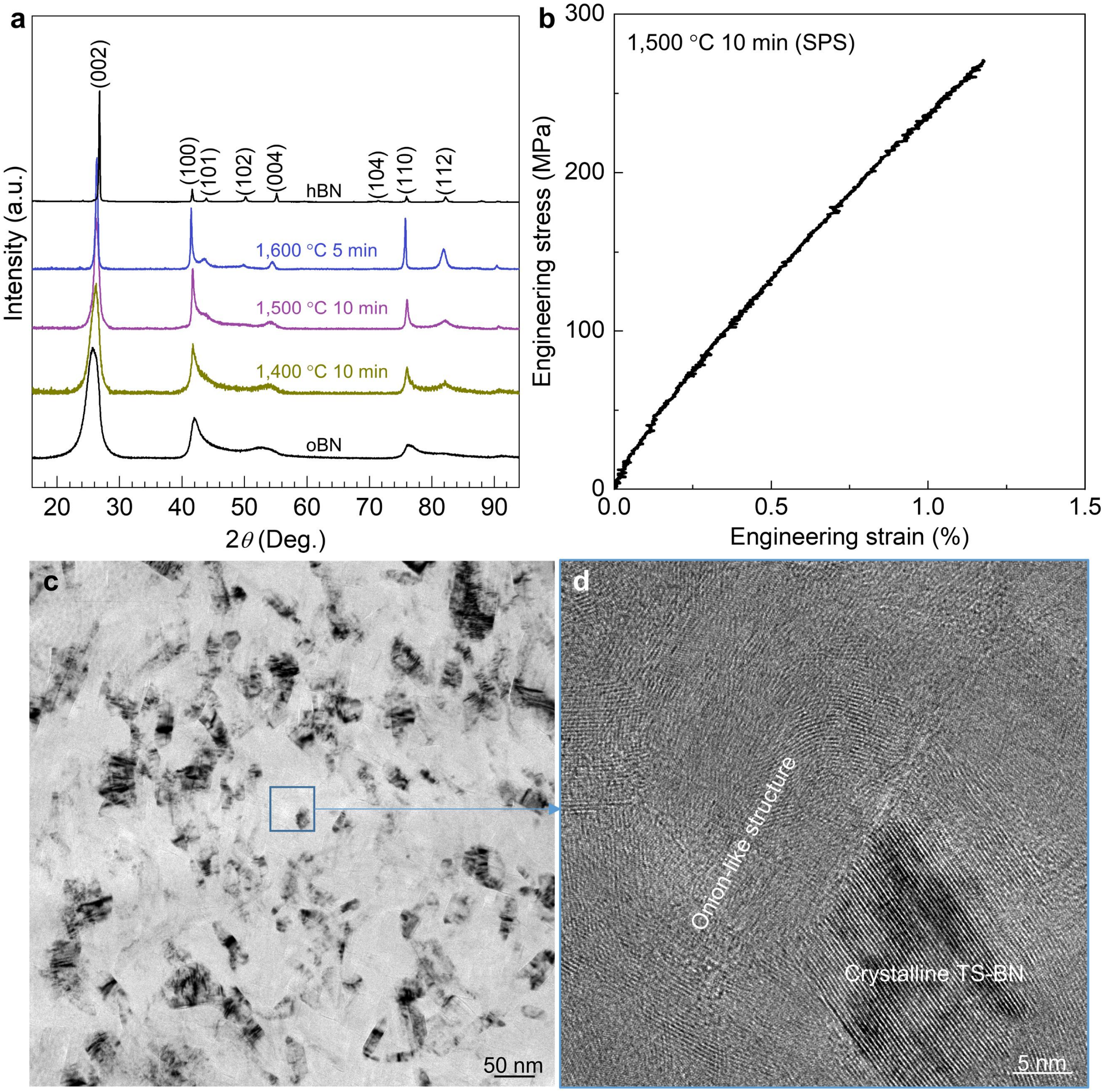

Supplementary Figure 5 | Microstructure and performance of BN ceramic obtained through SPS at 1,500 °C for 10 min. a, XRD patterns of ceramic samples obtained by sintering oBN at 1,400 °C, 1,500 °C, and 1,600 °C. **b**, Uniaxial compressive stress–strain curve of the 1,500 °C-sintered sample. **c** and **d**, TEM and HRTEM images of 1,500 °C-sintered sample. The crystalline TS-BN and residual untransformed onion-like BN structure can be observed in the sample.

Supplementary Table 1 | Peak positions (2θ), Miller indices, and relative intensities of simulated diffraction peaks of hypothetical twist-stacked BN structures. The XRD patterns are shown in Extended Data Fig. 5.

Structure	2θ (Deg.)	Miller indices	Relative intensity	2θ (Deg.)	Miller indices	Relative intensity	2θ (Deg.)	Miller indices	Relative intensity
hBN	26.75	(0 0 2)	100.0	44.1	(1 0 1)	5.3	55.15	(0 0 4)	7.1
	41.85	(1 0 0)	16.4	50.35	(1 0 2)	15.8			
38.2-I	25.70	(0 0 2)	100.0	43.93	(2 1 1)	13.8	52.86	(0 0 4)	7.5
	41.85	(2 1 0)	8.3	49.76	(2 1 2)	8.4			
38.2-II	26.13	(0 0 4)	100.0	43.99	(2 1 2)	2.4	53.75	(0 0 8)	7.4
	41.84	(2 1 0)	7.3	46.57	(2 1 3)	5.8	54.18	(2 1 5)	3.2
	42.39	(2 1 1)	8.3	50.00	(2 1 4)	7.3			
38.2-III	26.34	(0 0 6)	100.0	44.03	(2 1 3)	3.8	52.87	(2 1 7)	2.8
	41.84	(2 1 0)	7.7	45.67	(2 1 4)	1.0	54.22	(0 0 12)	7.3
	42.09	(2 1 1)	6.6	47.71	(2 1 5)	3.9	55.92	(2 1 8)	0.3
	42.82	(2 1 2)	1.2	50.12	(2 1 6)	7.6			
38.2-IV	26.40	(0 0 8)	100.0	44.03	(2 1 4)	2.5	52.19	(2 1 9)	2.8
	41.84	(2 1 0)	7.5	45.23	(2 1 5)	1.8	54.35	(0 0 16)	7.3
	41.98	(2 1 1)	6.6	48.30	(2 1 7)	3.8	56.78	(2 1 11)	0.7
	43.08	(2 1 3)	2.1	50.15	(2 1 8)	7.5			
38.2-V	26.40	(0 0 10)	100.0	44.03	(2 1 5)	3.0	51.77	(2 1 11)	2.9
	41.84	(2 1 0)	7.6	44.97	(2 1 6)	1.0	54.45	(0 0 20)	7.3
	41.93	(2 1 1)	6.3	46.06	(2 1 7)	0.6	55.34	(2 1 13)	0.3
	42.20	(2 1 2)	0.2	47.29	(2 1 8)	0.1	57.28	(2 1 14)	0.3
	42.64	(2 1 3)	0.9	48.66	(2 1 9)	3.4			
	43.26	(2 1 4)	1.1	50.16	(2 1 10)	7.6			
38.2-VI	26.46	(0 0 12)	100.0	44.04	(2 1 6)	2.5	51.53	(2 1 13)	3.0
	41.84	(2 1 0)	7.6	44.82	(2 1 7)	1.4	54.45	(2 1 15)	0.3
	41.90	(2 1 1)	6.4	46.68	(2 1 9)	0.7	54.48	(0 0 24)	7.3
	42.40	(2 1 3)	0.9	48.93	(2 1 11)	3.4	57.67	(2 1 17)	0.4
	43.38	(2 1 5)	1.4	50.19	(2 1 12)	7.5			
27.8-I	25.73	(0 0 2)	100.0	43.94	(3 1 1)	13.9	52.89	(0 0 4)	7.5
	41.85	(3 1 0)	8.3	49.78	(3 1 2)	8.4			
27.8-II	26.14	(0 0 4)	100.0	43.99	(3 1 2)	2.4	53.69	(0 0 8)	7.4
	41.84	(3 1 0)	7.3	46.56	(3 1 3)	5.8	54.15	(3 1 5)	3.2
	42.39	(3 1 1)	8.3	49.98	(3 1 4)	7.3			
27.8-III	26.32	(0 0 6)	100.0	44.02	(3 1 3)	3.8	52.85	(3 1 7)	2.8
	41.84	(3 1 0)	7.6	45.66	(3 1 4)	1.0	54.18	(0 0 12)	7.3
	42.09	(3 1 1)	6.6	47.70	(3 1 5)	3.9	55.90	(3 1 8)	0.3
	42.82	(3 1 2)	1.2	50.11	(3 1 6)	7.6			
27.8-IV	26.43	(0 0 8)	100.0	44.04	(3 1 4)	2.5	52.22	(3 1 9)	2.8
	41.84	(3 1 0)	7.6	45.24	(3 1 5)	1.8	54.42	(0 0 16)	7.3
	41.98	(3 1 1)	6.6	48.32	(3 1 7)	3.8	56.81	(3 1 11)	0.7
	43.09	(3 1 3)	2.1	50.17	(3 1 8)	7.5			
27.8-V	26.43	(0 0 10)	100.0	44.04	(3 1 5)	3.0	51.79	(3 1 11)	2.8
	41.84	(3 1 0)	7.6	44.98	(3 1 6)	0.9	54.42	(0 0 20)	7.3
	41.93	(3 1 1)	6.3	46.07	(3 1 7)	0.6	55.36	(3 1 13)	0.3
	42.20	(3 1 2)	0.2	47.30	(3 1 8)	0.1	57.31	(3 1 14)	0.3
	42.64	(3 1 3)	0.9	48.67	(3 1 9)	3.5			
	43.26	(3 1 4)	1.1	50.17	(3 1 10)	7.6			
27.8-VI	26.46	(0 0 12)	100.0	44.03	(3 1 6)	2.5	51.50	(3 1 13)	2.9
	41.83	(3 1 0)	7.6	44.80	(3 1 7)	1.3	54.41	(0 0 24)	7.3
	41.89	(3 1 1)	6.3	46.66	(3 1 9)	0.7	54.42	(3 1 15)	0.4
	42.39	(3 1 3)	1.0	48.90	(3 1 11)	3.5	57.63	(3 1 17)	0.4
	43.37	(3 1 5)	1.5	50.16	(3 1 12)	7.5			

Supplementary Table 2 | Strength and strain of traditional bulk ceramics under uniaxial compression at room temperature. Young's moduli of traditional ceramics were also listed.

Ceramic	Compressive strength (MPa)	Young's modulus (GPa)	Elastic strain (%)	Plastic strain (%)	Total strain (%)	Reference
CeO_2-PSZ	1,717		0.83	0.47	1.3	51
Mg-PSZ	1,860		0.9	1.2	2.1	25
MgO	833	270	0.31*	0.038	0.348	52
MgO	1,667	330	0.505*	0.041	0.546	
MgO	627		0.595		0.595	53
Al_2O_3	2,100		0.524		0.524	54
Al_2O_3	3,000	370	0.81*		0.81	55
Al_2O_3	4,000	406	0.99*		0.99	
Al_2O_3	2,600	350	0.74*		0.74	
AlN	1,970	302	0.652*	0.061	0.713	56
AlN	2,700	348	0.776*	0.083	0.859	
AlN	4,350		1.4		1.4	57
AlN	2,877		0.836		0.836	
Si_3N_4	2,000	280	0.714*		0.714	55
Si_3N_4	3,500	310	1.129*		1.129	
Si_3N_4	5,500	297	1.852*	0.169	2.021	58
SiC	3,734		0.805		0.805	24
SiC	3,987		0.866		0.866	
SiC	5,937		1.182		1.182	
SiC	6,115	460	1.257		1.257	
B_4C	2,900	445	0.652		0.652	59
B_4C	4,500	464	0.97		0.97	
B_4C	6,200	464	1.336		1.336	
B_4C	2,583	362	0.714*	0.058	0.772	60
B_4C	5,687	472	1.205*	0.124	1.329	
Diamond	8,680	700	1.24*		1.24	55
Diamond	16,530	1,200	1.378*		1.378	
Diamond	6,900	953	0.724*		0.724	
Diamond	4,500	800	0.563*		0.563	
Diamond	5,800	925	0.627*		0.627	
Diamond	10,000	1,050	0.952*	0.241	1.193	61
Diamond	20,000	1,210	1.653*	0.267	1.92	
Ti_2AlC	912		0.96		0.96	62
Ti_2AlC	1,027		1.0	0.13	1.13	63
Ti_2AlC	606		0.69	0.18	0.87	
Ti_2AlC	1,123				0.92	64
Cr_2AlC	1,160				0.61	65
Cr_2AlC	985				0.52	
Ti_2SC	1,400				0.45	66
SiO_2	690	73	0.94*		0.94	67
SiO_2	1,380	73	1.89*		1.89	
Graphite	78	11			2.1	68
hBN	112		1.1	1.1	2.2	16
hBN	82				1.9	

*Elastic strain estimated from the formula $\varepsilon = \sigma/E$, where ε was elastic strain, σ and E were the compressive strength and Young's modulus obtained from experiments, respectively.

Supplementary Table 3 | Compressive strength (MPa), fracture strain (%), flexural strength (MPa), tensile strength (MPa), Young's moduli (GPa) and fracture toughness K_{IC} (MPa·m$^{1/2}$) of TS-BN and hBN ceramics.

Samples	Compressive strength±s.d. (n=5)	Fracture strain±s.d. (n=5)	Flexural strength±s.d. (n=5)	Tensile strength±s.d. (n=5)	Young's modulus±s.d. (n=5)	K_{IC}±s.d. (n=5)
TS-BN[a]	626.2±12.3	13.6±0.58	199.2±8.7	59.1±4.9	54±4.8	1.96±0.17
hBN[b]	135.2±4.2	2.1±0.26	60.8±3.7	14.8±1.2	58±5.1	0.78±0.13
hBN[c]	57.4±3.8	0.6±0.14	22.0±1.9	15.3±1.5	61±5.6	0.39±0.11

[a] TS-BN ceramic sintered by SPS at 1,600 °C for 5 min.

[b] Home-made hBN ceramic prepared by sintering hBN nanosheets at 1,800 °C for 10 min.

[c] Commercial hBN ceramic.

Error bars indicate 1 s.d. (n=5 for compressive strength, fracture strain, flexural strength, tensile strength, Young's moduli, and K_{IC}).

Supplementary Table 4 | Compressive strength (MPa) and fracture strain (%) of TS-BN ceramics synthesized by SPS and hot-pressing (HP) sintering under varying conditions.

Samples	T (°C)/Time (min)/ Sintering method	Compressive strength±s.d. (n=5)	Fracture strain±s.d. (n=5)
TS-BN	1,600 / 5 / SPS	626.2±12.3	13.6±0.58
TS-BN	1,600 /10 / SPS	453.9±13.5	10.7±0.45
TS-BN	1,700 / 10 / SPS	332.3±9.1	4.1±0.27
TS-BN	1,650 / 5 / HP	772.5±14.7	10.2±0.66
TS-BN	1,750 / 5 / HP	545.6±9.2	6.7±0.47
TS-BN	1,850 / 5 / HP	350.7±7.9	6.1±0.62

Error bars indicate 1 s.d. (n=5).

Supplementary Table 5 | DFT-calculated cleavage energy (*E*c), slipping energy (*E*s), in-plane Young's modulus (*Y*) along the slipping direction, and the resulting deformability factor (Ξ) of hypothetical θ-tBN crystals with twist-stacked structure.

Twist angle (θ, °)	0	38.2	27.8	17.9	11.6
Cleavage energy (E_c, eV/atom)	0.0210	0.0225	0.0225	0.0224	0.0225
Slipping energy (E_s, eV/atom)	6.92×10^{-3}	8.7×10^{-5}	8.8×10^{-5}	11.8×10^{-5}	6.3×10^{-5}
Young's modulus (*Y*, GPa)	851.5	820.8	821.2	820.6	819.4
Deformability factor (Ξ, 1/GPa)	0.00356	0.3140	0.3118	0.2309	0.4355

References

51. Tsai, J. F., Chon, U., Ramachandran, N. & Shetty, D. K. Transformation plasticity and toughening in CeO_2 - partially - stabilized zirconia–alumina (Ce-TZP/Al_2O_3) composites doped with MnO. *J Am. Ceram. Soc.* **75**, 1229-1238 (1992).
52. Cotton, J. The property data of "*Magnesia-magnesium oxide (MgO) properties & applications*". AZoM, https://www.azom.com/article.aspx?ArticleID=54 (2019)
53. Crampon, J. & Escaig, B. Mechanical properties of fine-grained magnesium oxide at large compressive strains. *J Am. Ceram. Soc.* **63**, 680-686 (1980).
54. Shackelford, J. F. *Introduction to materials science for engineers.* (Prentice Hall, Englewood Cliffs, NJ, 1996).
55. Matweb, Material property data, https://www.matweb.com/index.aspx (2022)
56. Davey, R. The property data of "*Aluminium nitride / aluminum nitride (AlN) - Properties and applications*". AZoM, https://www.azom.com/article.aspx?ArticleID=610 (2022)
57. Subhash, G. & Ravichandran, G. Mechanical behaviour of a hot pressed aluminum nitride under uniaxial compression. *J Mater. Sci.* **33**, 1933-1939 (1998).
58. Sorrell, C. The property data of "*Silicon nitride (Si_3N_4) properties and applications*". AZoM, https://www.azom.com/article.aspx?ArticleID=53 (2001)
59. Blumenthal, W. R. "*High strain rate compression testing of ceramics and ceramic composites*" in J. J. Swab Ed., Advances in Ceramic Armor. *Ceram. Eng. Sci. Proc.* **26**, 89-96 (2005).
60. Cotton, J. The property data of "*Boron carbide (B_4C) - Properties and information about boron carbide*". AZoM, https://www.azom.com/article.aspx?ArticleID=75 (2001)
61. Baily, S. The property data of "*Diamond (C) - Properties and applications*". AZoM, https://www.azom.com/article.aspx?ArticleID=262 (2001)
62. Poon, B., Ponson, L., Zhao, J. & Ravichandran G. Damage accumulation and hysteretic behavior of MAX phase materials. *J Mech. Phys. Solids* **59**, 2238-2257 (2011).
63. Benitez, R. *et al.* Mechanical properties and microstructure evolution of Ti_2AlC under compression in 25-1100°C temperature range. *Acta Mater.* **189**, 154-165 (2020).
64. Hu, L. *et al.* High-performance metal/carbide composites with far-from-equilibrium compositions and controlled microstructures. *Sci. Rep.* **6**, 35523 (2016).
65. Tian, W., Sun, Z.M., Hashimoto, H. & Du, Y. Compressive deformation behavior of ternary compound Cr_2AlC. *J Mater. Sci.* **44**, 102-107 (2009).
66. Amini, S., Barsoum, M. W. & El-Raghy, T. Synthesis and mechanical properties of fully dense Ti_2SC. *J Am. Ceram. Soc.* **90**, 3953-3958 (2007).
67. Murray, L. The property data of "*Silica - Silicon Dioxide (SiO_2)*". AZoM, https://www.azom.com/article.aspx?ArticleID=1114 (2001)
68. Seldin, E. J. Stress-strain properties of polycrystalline graphites in tension and compression at room temperature, *Carbon* **4**, 177-191 (1966).
